# Examination of the Effects of Curvature in Geometrical Space on Accuracy of Scaling Derived Projections of Plant Biomass Units: Applications to the Assessment of Average Leaf Biomass in Eelgrass Shoots

**DOI:** 10.1155/2019/3613679

**Published:** 2019-04-23

**Authors:** Héctor Echavarría-Heras, Cecilia Leal-Ramírez, Enrique Villa-Diharce, Abelardo Montesinos-López

**Affiliations:** ^1^Centro de Investigación Científica y de Estudios Superiores de Ensenada, Carretera Ensenada-Tijuana No. 3918, Zona Playitas, Código Postal 22860, Apdo. Postal 360 Ensenada, B.C., Mexico; ^2^Centro de Investigación en Matemáticas, A.C. Jalisco s/n, Mineral Valenciana, Guanajuato Gto., Código Postal 36240, Mexico; ^3^Departamento de Matemáticas, Centro Universitario de Ciencias Exactas e Ingenierías (CUCEI), Universidad de Guadalajara, 44430 Guadalajara, Jalisco, Mexico

## Abstract

Conservation of eelgrass relies on transplants and evaluation of success depends on nondestructive measurements of average leaf biomass in shoots among other variables. Allometric proxies offer a convenient way to assessments. Identifying surrogates via log transformation and linear regression can set biased results. Views conceive this approach to be meaningful, asserting that curvature in geometrical space explains bias. Inappropriateness of correction factor of retransformation bias could also explain inconsistencies. Accounting for nonlinearity of the log transformed response relied on a generalized allometric model. Scaling parameters depend continuously on the descriptor. Joining correction factor is conceived as the partial sum of series expansion of mean retransformed residuals leading to highest reproducibility strength. Fits of particular characterizations of the generalized curvature model conveyed outstanding reproducibility of average eelgrass leaf biomass in shoots. Although nonlinear heteroscedastic regression resulted also to be suitable, only log transformation approaches can unmask a size related differentiation in growth form of the leaf. Generally, whenever structure of regression error is undetermined, choosing a suitable form of retransformation correction factor becomes elusive. Compared to customary nonparametric characterizations of this correction factor, present form proved more efficient. We expect that offered generalized allometric model along with proposed correction factor form provides a suitable analytical arrangement for the general settings of allometric examination.

## 1. Introduction

The model of relative growth of Huxley [1] is formally stated by means of a scaling relationship of the form

(1) $$\begin{array}{c} w=\beta a^{\alpha }, \end{array}$$

where *w* and *a* are measurable traits and the parameter *α* is designated as the allometric exponent, while *β* is identified as the normalization constant. This model, also termed equation of simple allometry, has been extensively used in research problems in biology [1–5], physics [6], economics [7], earth sciences [8], resource management, and conservation [9, 10], among other fields.

Eelgrass provides nursery for waterfowl and fish species. By trapping sediment and stumping wave energy, this seagrass promotes shoreline stabilization. Eelgrass services also include nutrient recycling, water filtration, and carbon dioxide removal. Current anthropogenic influences threaten eelgrass permanence. Conservation efforts rely on plot transplanting in a fundamental way. Monitoring effectiveness depends on measurements of standing stock and productivity through time. This makes the assessment of average leaf biomass in shoots a necessary input. But traditional estimation of eelgrass leaf biomass units relies on destructive methods. This could alter shoot density in a developing transplant. Thus evaluation renders indirect assessment methods necessary [10, 11]. Results show that an allometric scaling of the form of (1) for eelgrass leaf biomass *w* and associated area *a* is consistent [11]. Derived projections of individual leaf biomass convey useful surrogates for mean leaf biomass in shoots. Moreover, estimates of the parameters *α* and *β* are invariant within a given geographical region [10–12]. Hence, estimates fitted at site can endow suitable projections of leaf biomass values currently observed in other places of the region. This bears the referred allometric projections of a convenient nondestructive feature ([11, 13]).

Simplicity of (1) makes allometric projection of eelgrass leaf biomass units convenient. But there are caveats on dependability. For instance, even though *α* and *β* are invariant, environmental influences can induce a relative extent of variability on local estimates ([12, 14]). Besides, the response in the power function-like scaling of (1) is very sensitive to variation of parameter estimates. Then, accuracy of derived proxies is subject of error propagation of estimates. In addition, there are factors of biological scaling which can influence precision of estimates (e.g., [10, 11, 13, 15]). Packard [16] questioned results of Mascaro et al. [17] on allometric examination, and Mascaro et al. [18] responded to criticism. Going over this exchange highlights the relevance of procedural factors in determining precision of parameter estimates of allometric scaling. It also offers a convenient framework for the aims of the present research.

An important factor influencing precision of estimates of allometric parameters is analysis method. A widespread approach is the traditional analysis method of allometry (TAMA hereafter). It relies on a log transformation of data in arithmetical scale in order to contemplate a linear regression model in geometrical scale. Then, the fitted line is back-transformed to yield a two-parameter power function in the original scale. But embracing this procedure fuels a vivid unresolved debate. Views assert that this protocol can lead to biased results (e.g., [16, 19–29]). And other practitioners consistently wed to the idea that logarithmic transformations are necessary (e.g., [18, 30–39]). An alternative to the TAMA approach is using nonlinear regression methods in the direct scale of the data [16]. Echavarria-Heras et al. [11] concluded that producing allometric projections of average leaf biomass in eelgrass shoots must rely on this protocol. Yet a direct nonlinear regression approach in allometry is also not unfailing. For instance, inadequate identification of the inherent error structure can lead to significant bias [18]. Besides, Lai et al. [30] found that estimates of allometric parameters fitted by nonlinear regression can exhibit a high sensitivity to the largest values of the covariate. Therefore, evaluation of analysis method suitability in acquiring consistent eelgrass leaf biomass proxies needs revision.

The adoption of methods of curvature in geometrical space could offer a way to overcome inadequacy of the TAMA procedure ([27, 40–42]). In particular, it is pertinent to examine if taking curvature into account leads to improved accuracy of eelgrass leaf biomass proxies. But, according to Mascaro et al [18], curvature could manifest because of methodological factors of data gathering. Thus, an examination of the effect of curvature in eelgrass leaf biomass allometry must also take into account a possible participation of data quality effects. Mascaro et al. [18] reminds on three ways of handling curvilinearity in geometrical space. One is by separating data to contemplate different local linear models to account for heterogeneity of effects of the covariate [43–45]. A second one is by fitting a polynomial model [46–49]. A third approach endorses direct nonlinear regression assuming a heteroscedastic error structure as contemplated by Mascaro et al. [17]. Either approach above bears complexity beyond the linear model in geometrical space that associates with the customary bivariate power function of allometry. This suggested putting forward a generalized allometric model intended to deal with curvature in geometrical space. This paradigm incorporates parameters that change as continuous functions of the log transformed covariate ([27, 39, 46]). As we explain further on, the curvature arrangements recommended by Mascaro et al. [18] can be all derived from the offered formalization. Moreover, a nonzero intercept power function that Packard [50] recommends to handle curvilinearity in geometrical space also derives from the presented generalized scaling model.

But any scheme addressing curvature in geometrical space depends on a factor for correction of bias of retransformation of the regression error. In the general settings, if *ϵ* stands for the regression error, then the said correction factor, through denoted using the symbol *δ*(*ϵ*), is given by the mean of the exponentiated error random variable; that is, *δ*(*ϵ*) = *E*(*e*^∈^) [51–53]. Furthermore, the TAMA approach relies on the essential assumption that *ϵ* is additive, normally distributed, and homoscedastic [33]. When this happens, *δ*(*ϵ*) takes its lognormal-mean form [51–53]. But if *ϵ* fails to be normally distributed, there are two possibilities. If the distribution of *ϵ* is known, we could derive a closed form for *δ*(*ϵ*). In turn, if the error distribution is not identified a priori, a widespread approach is taking *δ*(*ϵ*) in the nonparametric form given by the smearing estimator of bias of Duan [54]. Still, there are provisions on this. A smearing estimate form can fail to compensate the downward retransformation bias of logged data ([53, 55, 56]). Thus, in a circumstance where *ϵ* is unspecified, characterizing *δ*(*ϵ*) seems elusive. Here, we put forward an arrangement for *δ*(*ϵ*) aimed to get around this circumstance. Zeng and Tang [57] proposed a nonparametric alternate to the smearing form. It matches the first three terms' partial sum of a power series expression of *E*(*e*^∈^), assuming *E*(*ϵ*) = 0. Form suggested here corresponds to a generalization of this construct. It does not abide the restriction *E*(*ϵ*) = 0 and matches an *n*  −terms partial sum approximation of the exponential series representation of *E*(*e*^∈^). The partial sum that maximizes reproducibility strength of retransformed mean response sets criterion to choose *n*.

Present results show that a consideration of curvature in geometrical space, as well as a suitable characterization of the correction factor of retransformation bias, offers consistent allometric proxies of observed mean leaf biomass in eelgrass shoots. Hence, contrary to views asserting direct nonlinear regression as mandatory in allometric examination, our findings validate a parallel reliability of log transformation based methods. This is well in line with claims of Mascaro et al. [18] and many others about blaming the use of logarithms of incongruent results in allometric analysis. Moreover, keeping the analysis in geometrical space unraveled heterogeneity in the inherent leaf biomass scaling pattern. This could not be achieved by clinging to direct nonlinear regression in arithmetic space as the only valid approach of allometric examination. Offered analytical arrangement is expected to be applicable in the general settings of allometry.

## 2. Materials and Methods

### 2.1. Data

For the aims of the present research, we relied on an extensive eelgrass data set collected in San Quintin Bay, a coastal lagoon on the Pacific side of the Baja California Peninsula, México (30°30' N – 116°00′W), and through a 13 months' long sampling period covering a whole-year cycle. Data composes measurements of length (mm), width (mm), and dry weight *w* (g) of a total of 10412 individual eelgrass leaves taken from 20 randomly thrown 400cm^2^ quadrats every monthly visit to the site. A sampling visit will be further referred to as “sampling time” in the text. The length times width proxy [11] provided estimations of leaf area *a* (mm^2^). In order to test for methodological influences of data gathering, we processed raw data set according to a mean plus or minus two standard deviations outlier's removal procedure [58].  Appendix A presents results of an exploratory analysis of data.

### 2.2. Models

As above specified symbols, *w* and *a* stand for the biomass of an individual eelgrass leaf and its respective area one to one. Echavarría-Heras et al. [11] assert that these variables can be related through the bivariate allometric model of (1). One procedure to acquire estimates for the parameters *α* and *β* is fitting directly in arithmetical scale a nonlinear homoscedastic regression model. Besides, we can use a TAMA approach, that is, fitting the linear regression model

(2) $$\begin{array}{c} v=\ln\beta +\alpha u+\epsilon , \end{array}$$

where *v* = ln⁡*w*, *u* = ln⁡*a*, and *ϵ* is a random error term assumed to be normally distributed with zero mean and variance *σ*^2^, that is, *ϵ* ~ *N*(0, *σ*^2^).

We conceive curvature in geometrical space as a circumstance where fitting results of regression model of (2) are inconsistent. Dealing with this situation amounts for considering complexity beyond incorporated by (1). One possible approach to address curvature is assuming that scaling parameters *α* and *β* in (2) depend continuously on the covariate ([27, 39, 46]). This is consistent with the generalized allometric model,

(3) $$\begin{array}{c} w=\beta \left(\;a\;\right)a^{\alpha \left(a\right)}, \end{array}$$

with *β*(*a*) and *α*(*a*) intended to be continuous and differentiable functions defined on *R*^+^ and with *β*(*a*) being positive. Certainly a log transformation *v* = ln⁡*w*, *u* = ln⁡*a* of (3) establishes the regression model

(4) $$\begin{array}{c} v=v_{C}\left(\;a,u\;\right)+\epsilon , \end{array}$$

where

(5) $$\begin{array}{c} v_{C}\left(\;a,u\;\right)=\ln\beta \left(\;a\left(\;u\;\right)\;\right)+\alpha \left(\;a\left(\;u\;\right)\;\right)u, \end{array}$$

where *ϵ*, a residual error term, is conceived as a random variable that in the general settings is *ψ*-distributed with mean *μ* and variance set by a function *σ*^2^(*u*) of the covariate *u*, that is, *ϵ* ~ *ψ*(*μ*, *σ*^2^(*u*)).

Setting *β*(*a*(*u*)) = *β* and *α*(*a*(*u*)) = *α* with *α* and *β* constants reduces (4) to the regression model of (2). In Appendix B, we explain that (3) accommodates all curvature paradigms suggested by Mascaro et al. [18]. These include a biphasic and a polynomial model in geometrical space, as well as the nonlinear heteroscedastic model referred to by Mascaro et al. [17] in direct arithmetical space. Moreover, as shown in Appendix B, the three-parameter power function chosen as an alternate standard for curvature [59] also derives from (3).

#### 2.2.1. Biphasic Model in Geometrical Space

In order to characterize the model of (3) in a biphasic mode, we let *β*(*a*) = *β*_*B*_(*a*) and *α*(*a*) = *α*_*B*_(*a*), such that

(6) αBa=∑12ϑiuaαiβBa=exp⁡∑12ϑiualn⁡βi

including parameters *β*_*i*_ and *α*_*i*_ and the function *ϑ*_*i*_(*u*(*a*)) given by

(7) $$\begin{array}{c} \vartheta_{i}\left(\;u\left(\;a\;\right)\;\right)=\left\{\begin{array}{cc} \left(2-i\right)\left(1-H\left(u-u_{b}\right)\right) & \text{for}\;\;i=1\\ \left(i-1\right)H\left(u-u_{b}\right) & \text{for}\;\;i=2, \end{array}\right. \end{array}$$

for *i* = 1,2. *H*(*u* − *u*_*b*_) is a Heaviside function *H*(*z*) [60], evaluated at *z* = *u* − *u*_*b*_ and correspondingly *u*_*b*_ = ln⁡*a*_*b*_, with *a*_*min*_ ≤ *a*_*b*_ ≤ *a*_*max*_ being a point separating growth phases *u* ≤ *u*_*b*_ and *u* > *u*_*b*_. Then, denoting by means of *v*_*B*_(*a*, *u*) the resulting form of *v*_*C*_(*a*, *u*) from (4), we get the biphasic regression model

(8) $$\begin{array}{c} v=v_{B}\left(\;a,u\;\right)+\epsilon , \end{array}$$

where *ϵ* is a random error term as defined in (4) and

(9) $$\begin{array}{c} v_{B}\left(\;a,u\;\right)=\overset{2}{\underset{1}{\sum }}\vartheta_{i}\left(\;u\left(\;a\;\right)\;\right)f_{i}\left(\;u\left(\;a\;\right)\;\right) \end{array}$$

with

(10) $$\begin{array}{c} f_{i}\left(\;u\left(\;a\;\right)\;\right)=\ln\beta_{i}+\alpha_{i}u\left(\;a\;\right), \end{array}$$

where *β*_*i*_ and *α*_*i*_ for *i* = 1,2 parameters are to be estimated from data.

#### 2.2.2. Polynomial Model in Geometrical Space

Similarly, assume that *α*(*a*) = *α*_*P*_(*a*) and *β*(*a*) = *β*_*P*_(*a*), with

(11) αPa=∑0nαkuakβPa=e∑0nβkuak.

*α*_*k*_ and *β*_*k*_ for 1 ≤ *k* ≤ *m* are coefficients; one can acquire a polynomial representation *v*_*P*_(*a*, *u*) for the generalized mean response function in geometrical space *v*_*C*_(*a*, *u*). This way the polynomial form of regression (4) becomes

(12) $$\begin{array}{c} {v=v}_{P}\left(\;a,u\;\right)+\epsilon , \end{array}$$

where *ϵ* is a random error term as defined in (4), with

(13) $$\begin{array}{c} v_{P}\left(\;a,u\;\right)=\overset{m}{\underset{0}{\sum }}{p_{k}u\left(a\right)}^{k}, \end{array}$$

and *p*_*k*_, for *k* = 0,2,…, *m* parameters.

#### 2.2.3. Nonlinear Heteroscedastic Model in Arithmetical Space

As we explain ahead, direct algebraic manipulation of (3) leads to the consideration of the nonlinear heteroscedastic regression model addressed by Mascaro et al. [17]; namely,

(14) $$\begin{array}{c} w={\beta_{\theta }a}^{\alpha_{\theta }}+a^{\theta }\epsilon , \end{array}$$

with *α*_*θ*_, *β*_*θ*_, and *θ* being parameters and *ϵ* being a zero mean normally distributed error term with *a* covariate dependent variance *σ*^2^(*a*) = *σ*^2^*a*^2*θ*^, that is, *ϵ* ~ *N*(0, *σ*^2^*a*^2*θ*^).

A nonlinear homoscedastic form derives from (14) by setting *θ* = 0; that is,

(15) $$\begin{array}{c} w=\beta_{o}a^{\alpha_{o}}+\epsilon , \end{array}$$

And, again *ϵ* is an additive error term assumed to be normally distributed with zero mean and variance *σ*^2^, that is, *ϵ* ~ *N*(0, *σ*^2^).

Appendix A deals with exploratory analysis of data Appendix B presents notation convention and also explains how all addressed paradigms derive from the generalized model of (3).  Appendix C explains the addressed forms of correction factor for bias of retransformation of the regression error. Fitting results of the geometrical space based models appear in Appendix D. Those corresponding to the nonlinear heteroscedastic and homoscedastic models pertain to Appendix E. Agreement between observed and projected values is commonly evaluated by analyzing values of Lin's Concordance Correlation Coefficient (CCC) [61]. This correspondence index is commonly denoted by means of the symbol *ρ*. Agreement will be defined as poor whenever *ρ* < 0.90, moderate for 0.90 ≤ *ρ* < 0.95, good for 0.95 ≤ *ρ* < 0.99, or excellent for *ρ* ≥ 0.99 [62]. Besides CCC values, we assessed reproducibility by comparing goodness-of-fit statistics, such as the coefficient of determination (CD), standard error of estimate (SEE), mean prediction error (MPE), total relative error (TRE), average systematic error (ASE), and mean percent standard error (MPSE) ([63–65]). For statistical tasks, we relied on the R package release 3.5.

## 3. Results

Exploratory analysis in Appendix A identifies maximum, minimum, and sample mean values for observed leaf area values *a* and associated dry weights *w*. We also explain distribution of variables in terms of quantiles of probability 0.1, 0.25, 0.50, 0.75, and 0.90, for both crude and processed data. Statistical exploration extends to log transformed values of these variables. We present Q-Q plots (quantile-quantile) for comparison of distribution patterns, as well as boxplots for the 13 months' long sampling scheme for both crude and processed data. We can learn that, from month 2 to month 6, a reduction in the values of weight and area occurred; this perhaps is explained by an increase in temperature during the period. We can be also aware that a similar variation pattern over time is shown in both raw and processed data sets.

### 3.1. Fitting Results of Geometrical Space Models

In order to validate curvature in geometrical space, we compared the linear model derived from (1) as well as biphasic and polynomial alternates derived from the generalized model of (3). Appendix B explains formal matters. Tables 1 and 2 summarize notation convention. Equations numbered beyond (15) belong to the appendices. Appendix D explains corresponding regression protocols.

**Figure 1:**
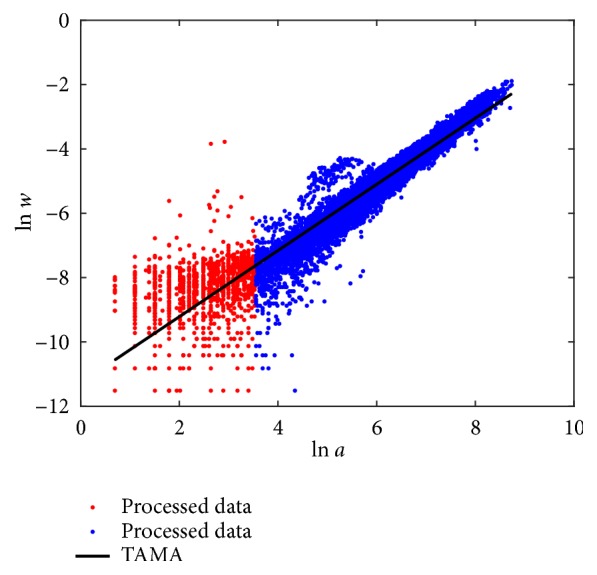
Spread about the TAMA's linear mean response function *E*_*T*_(*v* | *u*). Deviations about *E*_*T*_(*v* | *u*) shown by red dots suggest curvature.

Fitting results of the TAMA arrangement of (2) appear in Appendix D.  Figure 1 shows the spread about TAMA's linear mean response function *E*_*T*_(*v*∣*u*). We can visually ascertain that deviations from the linear mean response function *E*_*T*_(*v*∣*u*) suggest curvature (red dots). Thus, data processing removed inconsistent replicates but shown spread still deviates from a linear mean response. This suggests that curvature in geometrical space could not be explained by methodological factors related to data gathering.

**Figure 2:**
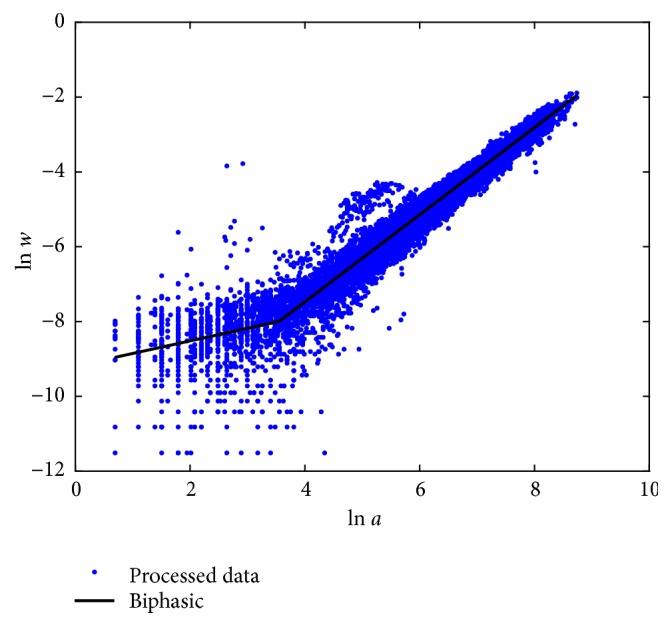
Spread about the biphasic mean response function *E*_*B*_(*v* | *u*). Compared with the plot in Figure 1, we observe that the biphasic choice offers a better account of variability of the log transformed response than the TAMA alternate.

Fitting results of the biphasic protocol of (8) are summarized in Appendix D.  Figure 2 displays the spread about mean response function *E*_*B*_(*v*∣*u*) in geometrical space. Compared with Figure 1, we can ascertain that the biphasic fit provides a consistent account of different variation patterns among smaller and larger leaves. We can visually ascertain that fit produced consistent results. This confirms a judgement that identified curvature might be due to intrinsic factors of leaf growth rather than methodological influences related to data gathering.

**Figure 3:**
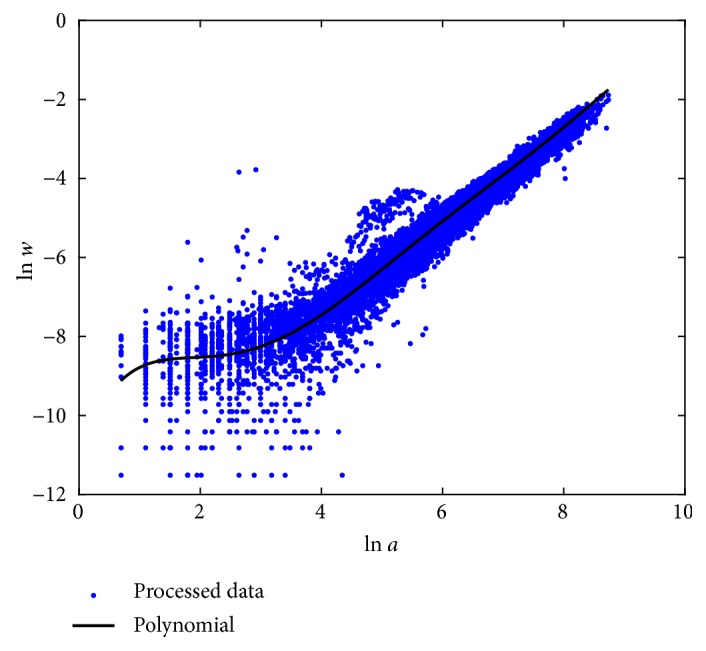
Spread about the polynomial mean response function *E*_*P*_(*v* | *u*). Compared with the plot in Figure 1, we can be aware that, as opposite to the TAMA protocol, the polynomial scheme offers a consistent account of curvature.

Appendix D presents fitting results of the polynomial model (*m* = 6).  Figure 3 displays dispersion about the polynomial mean response function in geometrical space *E*_*P*_(*v*∣*u*). A polynomial representation also exhibits higher consistency than the TAMA arrangement. Recalling the biphasic scheme, the polynomial suggests a smooth transition between two growing phases.

### 3.2. Model Selection in Geometrical Space

Assessment of models fitted on geometrical space relied on goodness-of-fit statistics, that is, the coefficient of determination, standard error of estimate, mean prediction error, total relative error, average systematic error, and mean percent standard error ([63–65]). Besides, we took into account concordance correlation coefficient [61] and Akaike's information index [66].  Table 3 presents results. Goodness-of-fit statistics and *ρ* and AIC values disfavored the TAMA protocol. On the contrary, comparison indices favored the biphasic model. Moreover, differences among indices but TRE and ASE for this scheme and the polynomial (*m* = 6) are slight. Particularly, the highest AIC is associated with the TAMA protocol (AIC = 15069.9, ∆AIC = 2491.9). Therefore, this model bears the less support. The biphasic choice delivered the smallest AIC's value (AIC = 12578.0, ∆AIC = 0). Nevertheless, difference in AIC is just barely relative to the *m* = 6 polynomial model, since this choice conveyed (AIC = 12615.0 and ∆AIC = 37). Thus, model confrontation shows that the TAMA protocol is unsuited, thus backing the assertion that whatever model aims to be consistent with the present data, it ought to be nonlinear in geometrical space.

### 3.3. Retransformation Results

The TAMA protocol was not supported by the model selection criteria. Anyway, for comparison, corresponding retransformation results are included in Appendix D. Related with the TAMA protocol, fitting results of the biphasic model display a relatively improved distribution of residuals about the zero line. Nevertheless, normal Q-Q plot still shows heavier tails than those expected for a normal distribution. And, again both test statistics and p values of an Anderson-Darling test [67] provide evidence against normality of residuals. This justifies choosing the nonparametric forms *δ*_*D*_(*ϵ*), *δ*_*ZT*_(*ϵ*), or *δ*_*n*_(*ϵ*) for compensation of downward bias induced by retransformation of the regression error ([11, 52, 53]).  Table 4 displays comparison statistics for the reproducibility strength of the biphasic mean response *E*_*Bδ*_(*w*∣*a*) as shaped by the different forms of *δ*(*ϵ*).

**Figure 4:**
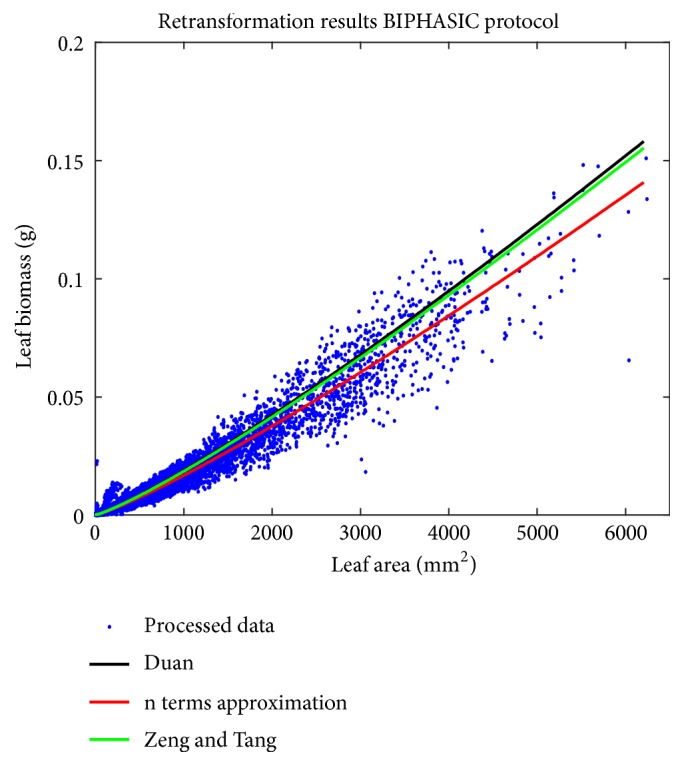
Spread about the biphasic mean response function *E*_*Bδ*_(*w* | *a*) in arithmetical space. Black lines relate to *δ*_*D*_(*ϵ*), green to *δ*_*ZT*_(*ϵ*), and red ones to *δ*_*n*_(*ϵ*).

We can learn that agreement between the biphasic mean response and leaf biomass data is best for *δ*_*n*_(*ϵ*). Figure 4 shows spread of processed leaf biomass values about the biphasic mean response function *E*_*Bδ*_(*w*∣*a*) as shaped by the considered forms of *δ*(*ϵ*). We can observe that both *δ*_*D*_(*ϵ*) and *δ*_*ZT*_(*ϵ*) overcompensate the bias correction by *δ*_*n*_(*ϵ*). Moreover results show that as opposed to the TAMA a biphasic protocol along with the *δ*_*n*_(*ϵ*) form offers consistent proxies of individual leaf biomass. But it is worth mentioning that in spite of the fact that model selection favored the biphasic scheme, examination of the polynomial model output reveals similar predictive strength to the biphasic alternate.

### 3.4. Assessing Curvature by Direct Nonlinear Regression

As suggested by Mascaro et al. [18], effects of curvature in geometrical space can be analyzed by means of the direct nonlinear heteroscedastic regression model of (14). In Appendix B, we explain that such a protocol also derives from the generalized bivariate allometric model of (3).  Table 5 presents pertinent notation convention. For comparison, we also present results for the associated homoscedastic case.

Fitting results of the heteroscedastic and homoscedastic models appear in Appendix E. We can learn that estimates for the normalization constant and scaling exponent parameters are very similar. Certainly, corresponding 95% confidence intervals display some overlap. As a result, we can expect similar reproducibility features for both models.  Table 6 presents comparison statistics.

**Figure 5:**
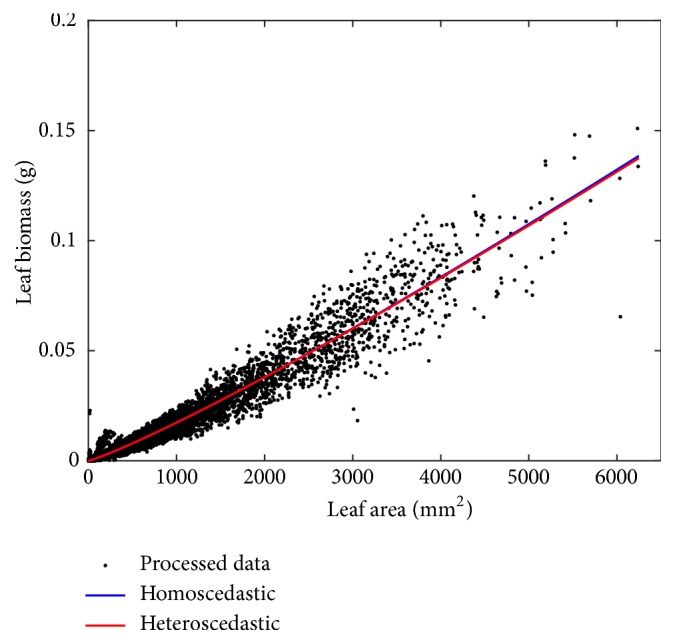
Spread about the mean response, curves *E*_*θ*_(*w* | *a*) and *E*_*o*_(*w* | *a*) associated with the nonlinear heteroscedastic and homoscedastic regression models of (14) and (15) one to one. The mean response *E*_*θ*_(*w* | *a*) is shown in black lines and those corresponding to *E*_*o*_(*w* | *a*) in red.

We can be aware that model assessment backs the heteroscedastic model. But selection here is mainly on qualitative grounds. It actually concerns the ability of the heteroscedastic model to identify an expected dependence of variance in the covariate. Certainly, the reproducibility strengths of both paradigms are equivalent. Indeed, Figure 5 shows that mean response curves *E*_*θ*_(*w*∣*a*) and *E*_*o*_(*w*∣*a*) differ just barely.

Results show that as it occurred for models fitted in geometrical space, data cleaning failed to correct a heavy tails problem for the nonlinear fits. This can be ascertained from the normal Q-Q plot of residuals. This strengthens our point on the consideration of a different error structure from the one assumed here. Exploring the effects of error structure in the fitting of models for curvature addressed here will be a matter of further research. Interestingly, both the homoscedastic and heteroscedastic models seem to induce the same reproducibility strengths.

### 3.5. Model Assessment in Arithmetical Space

The model selection assay in geometrical space summarized in Table 3 favored the biphasic protocol. Correspondingly, statistics in Table 6 support the nonlinear heteroscedastic model. Results of Table 4 endure *δ*_*n*_(*ϵ*) as required for largest reproducibility of retransformation output.  Table 7 allows assessment of these models. We can learn that half the number of comparison indices coincide (*ρ*, R^2^, SEE, and MPE). In addition, the biphasic model is favored by AIC, ASE, and MPSE. This sets criterion for selection of curvature in geometric space as a consistent paradigm for the present data. Accordingly, the biphasic model bears adequate.

### 3.6. Implications for Allometric Proxies of Mean Leaf Biomass in Eelgrass Shoots

We in turn consider allometric proxies for average leaf biomass in eelgrass shoots. In getting these surrogates, we aggregate allometric projections of individual leaf biomass conforming a shoot. For comparison, we consider individual leaf biomass surrogates produced by the different projection methods.  Table 8 compares resulting reproducibility strengths.

**Figure 6:**
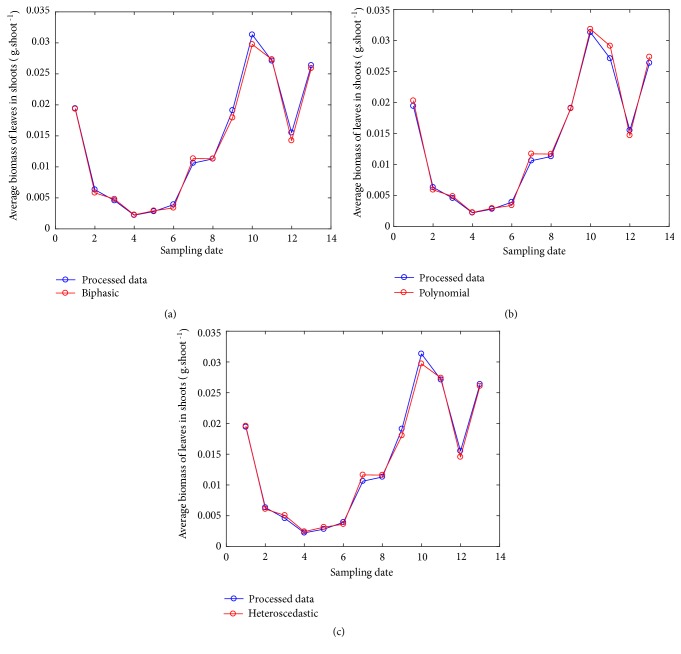
Average leaf biomass in shoots calculated from observed-processed data compared with their allometric projections. Projection lines resulting from the biphasic and polynomial models relied on the *δ*_*n*_(*ϵ*) form.

Results in [11] stablished that proxies derived from the TAMA protocol are inconsistent with observed values. This endorsed nonlinear regression in the direct scale as a requirement for reliability of allometric projections of mean leaf biomass in eelgrass shoots. But Table 8 shows that a curvature model fitted in geometrical space can offer proxies entailing similar predictive power to a nonlinear regression protocol. Plots in Figure 6 allow getting a glimpse of this assertion.

## 4. Discussion

**Figure 7:**
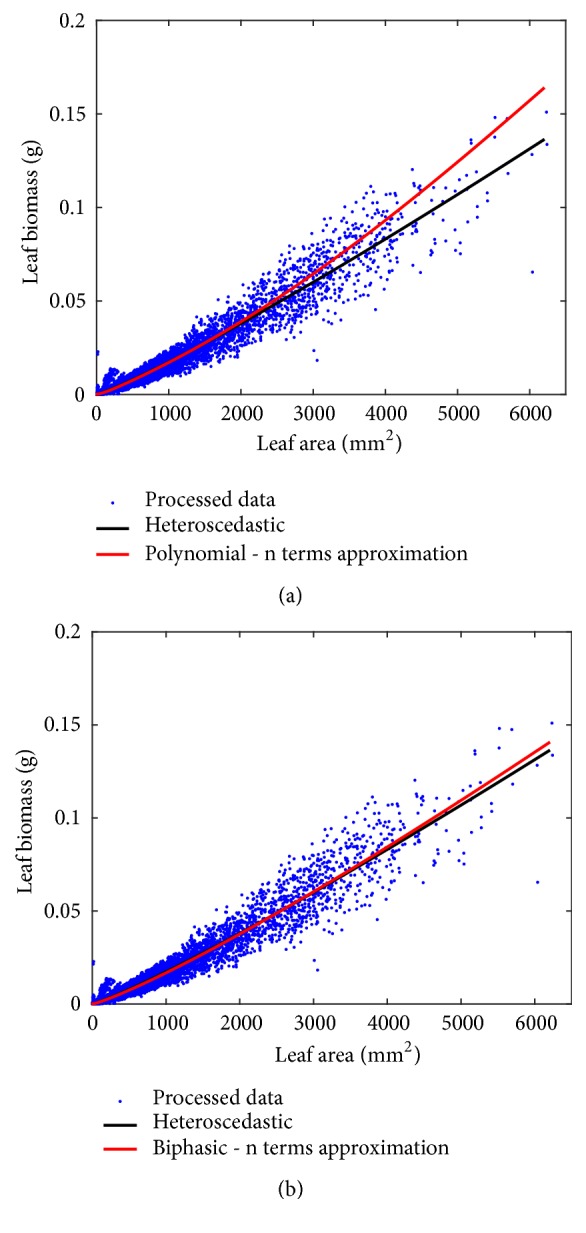
Trends of mean response functions calculated from the retransformed outputs of polynomial (a) and biphasic models (b) involving the *δ*_*n*_(*ϵ*) form of *δ*(*ϵ*). We can observe a differentiation in trends relative to that of a power function fitted in directly by means of the nonlinear heteroscedastic protocol of (14). Although relative deviations manifest for the biphasic model, differences in mean response patterns are more clearly depicted for the polynomial choice.

The customary bivariate allometric model of (1) offers nondestructive surrogates for average leaf biomass in eelgrass shoots [11]. But there are methodological factors that could influence dependability. Views assert that parameter identification based on logarithmic transformations leads to biased projections [20–29]. But other practitioners clung to this approach as meaningful and necessary in allometric examination [30–39]. This going over suggests that surpassing this controversy amounts to considering curvature in geometrical space. For that aim, we proposed the generalized model of (3). Approaches such as direct nonlinear heteroscedastic regression, as well as biphasic and polynomial protocols in geometrical space [18], became logical resultants from this construct. For present data model selection validated maintenance of the analysis in geometrical space. Nevertheless, at an empirical level, addressed protocols produced allometric projections of individual leaf biomass of correspondent precision. This was also verified for concomitant projections of average leaf biomass in shoots. But, from a qualitative standpoint, the nonlinear regression protocol mainly contributed by identifying expected dependence of the variance on covariate. Moreover.  Figure 7(a) depicts manifest differences in mean response trends between the polynomial fit and the nonlinear heteroscedastic model. Nonetheless, those in Figure 7(b) corresponding to this and the biphasic models differ but only barely. Then, a nonlinear regression scheme at best shaped a reasonable approximation of the mean response function resultant from curvature methods.

Differences in patterns of the biphasic and polynomial mean response functions relative to the nonlinear protocol exhibit that clinging to this last paradigm could impair detection of the true allometric relationship. Moreover, relying on direct nonlinear regression impairs identification of heterogeneity in the log transformed response as covariate changes. This further stresses on limitations of this device as a tool for allometric examination ([18, 30]). Oppositely, output of the selected biphasic model shown in Figure 2 suggests differentiation of growth patterns among smaller and larger leaves. Besides, the polynomial mean response in Figure 3 suggests a gradual transition between different growth phases. Thus, as opposed to direct nonlinear regression, a consideration of curvature in geometrical space could elucidate an inherent leaf growth pattern. This strengthens a judgement that the log transformation step, essential to traditional allometric examination, cannot be thrown away without losing relevant information ([33, 37]).

Mascaro et al. [18] conceived curvature in geometrical space as related to methodological factors of data gathering. But present examination corroborated consistency of curvature models for processed data. This suggests that manifestation of curvature is rather explained by intrinsic factors in leaf growth. Additionally, data processing failed to amend the heavy tails problem detected on Q-Q plots. This indicates departure of residuals from an assumed error structure. As a result, numerical values of the addressed correction factor forms turned out to be different, thus conveying ambiguity in selection of suitable mean response of models fitted in geometrical space. This could entail the only advantage of nonlinear regression method over log-transformation-curvature paradigms. But, also for this analysis method, an inadequate postulation of inherent error structure can lead to significant bias [18]. It seems then reasonable considering that a suitable characterization of error structure could lead to robustness of built allometric proxies, even when they derive from crude data. Steering to an error structure different from what is assumed here is a worthwhile subject of further research.

When dealing with similar data we suggest taking into account recommendations that come up from this examination. First, it is highly advisable to perform a preliminary examination of the spread around the straight line in geometrical space resulting from the model of (1). If further statistical exploration confirms that linearity and assumed error structure are consistent with data, Huxley's bivariate allometric model could suit. Otherwise, the arrangement of curvature, error structure, and correction factor form such as proposed here could be called into account for the analysis. The use of data cleaning procedures in order to achieve a better fit is controversial [11]. Instead of performing data processing a posteriori, it is highly advisable to rely on standardized data gathering procedures. This will prevent proliferation of inconsistent replicates that could exacerbate a heavy tails problem on Q-Q plots.

## 5. Conclusion

Failure to perform both a preliminary exploration of spread of log transformed allometric data and a sound evaluation of model adequacy could impair detecting a possible manifestation of curvature. As a consequence, the output of a traditional analysis method could set biased predictions of observed values. This circumstance could result in dismissal of a log transformation step in the analysis, giving way to contemplation of direct nonlinear regression as the only protocol to acquire reliable parameter estimates [11]. Results of this examination suggest that consideration of curvature in geometrical space as set by the model of (3) could offer dependable allometric proxies of average leaf biomass in eelgrass shoots.

From a general perspective, complexity as encompassed by the model of (3) can stand for curvature as conceived in allometric examination. Particularly, biphasic or polynomial protocols in geometrical space, as well as a direct nonlinear heteroscedastic regression model, derive as particular characterizations of this paradigm. Moreover all statistical models for accurate estimates of relative growth contemplated by Bervian et, al., [68] can be also accomodated by the present generalization of the model of simple allometry of Huxley. But empirical convenience on its own does not validate adoption of this paradigm as a general tool. Certainly, the Weierstrass approximation theorem [69] backs a polynomial regression model as a reasonable identification device for the generalized allometric model expressed in geometrical space. But suitability of retransformation results will sensibly depend on correction factor form. And a mean function resulting from a polynomial fitted in geometrical space will not enable characterization of functions *α*(*a*) and *β*(*a*) one to one. Furthermore, complexity of (3) could pose significant difficulties while attempting its identification through direct nonlinear regression methods. Needless to say, biological interpretation of the scaling functions *α*(*a*) and *β*(*a*) is also pending. A quest for efficient tools of nondestructive assessment of plant biomass units justifies addressing these examinations in a further research.

## Figures and Tables

**Figure 8:**
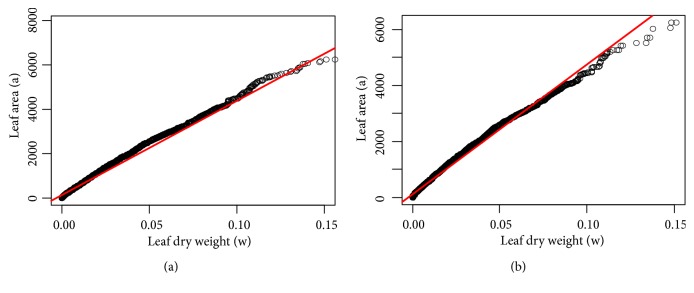
Q-Q plots (quantile-quantile) comparing distribution pattern of observations of eelgrass leaf area *a* and associated dry weight *w* for raw (a) and processed data (b).

**Figure 9:**
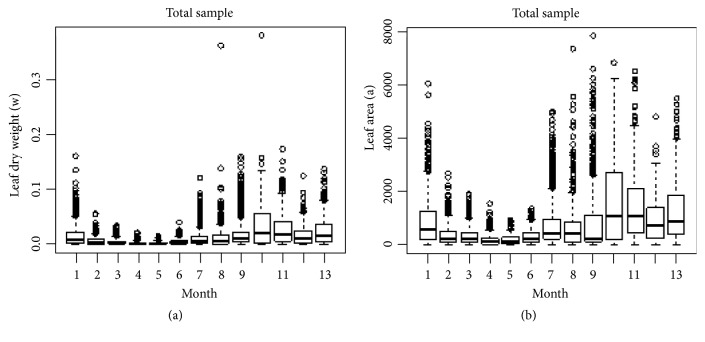
Boxplots for values of eelgrass leaf dry weight *w* (a) and linked area *a*(b) classified by month, as indicated in the horizontal axis. The data is from a sample of 10412 observations, before applying data processing.

**Figure 10:**
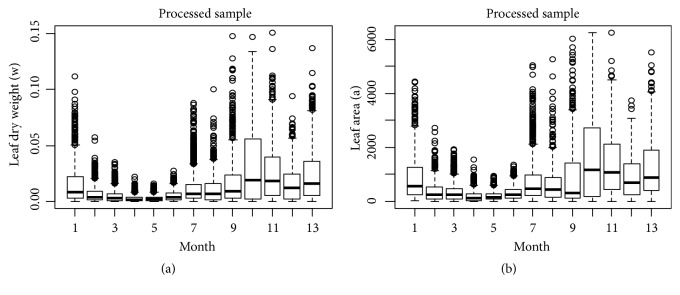
Boxplots for values of eelgrass leaf dry weight *w* (a) and linked area *a* (b) classified by month, as indicated in the horizontal axis. The data is from a sample of 10023 observations, remaining after applying an outlier removal procedure.

**Figure 11:**
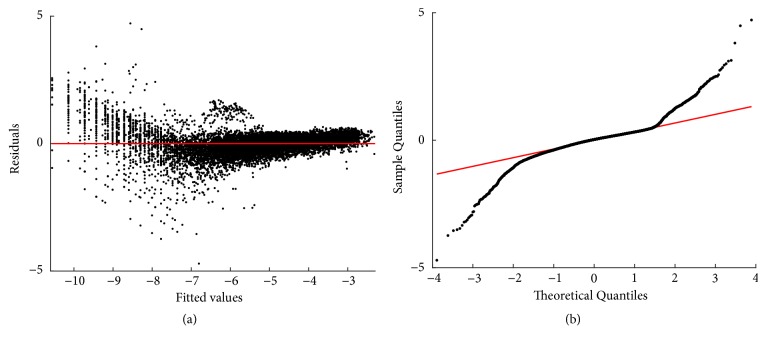
Residuals and normal probability plots ((a) and (b), resp.) resulting from fitting the linear regression model of the TAMA protocol (cf. (D.1) through (D.3)). A biased distribution of residuals around the zero line is depicted. Also, the normal Q-Q plot shows heavier tails than those expected for a normal distribution.

**Figure 12:**
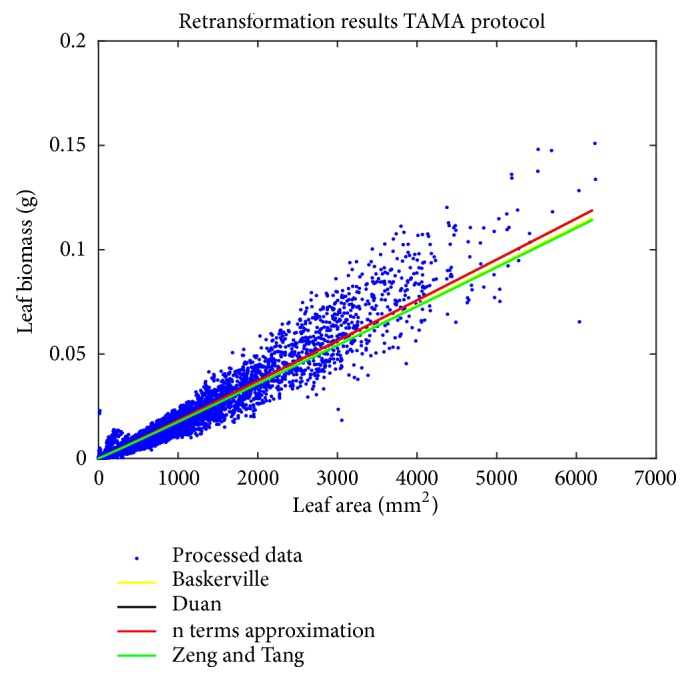
Spread about the mean response function *E*_*Tδ*_(*w* | *a*) obtained by retransforming the linear model of the TAMA protocol and using the addressed forms of the correction factor *δ*(*ϵ*) (Table 1). Black lines associated with *E*_*TD*_(*w* | *a*) green lines go with *E*_*TZT*_(*w* | *a*), and red ones go with *E*_*Tn*_(*w* | *a*) and in yellow we show those for *E*_*TB*_(*w* | *a*). We can learn of a biased spread of observed values about the mean response functions *E*_*Tδ*_(*w* | *a*) even after data processing.

**Figure 13:**
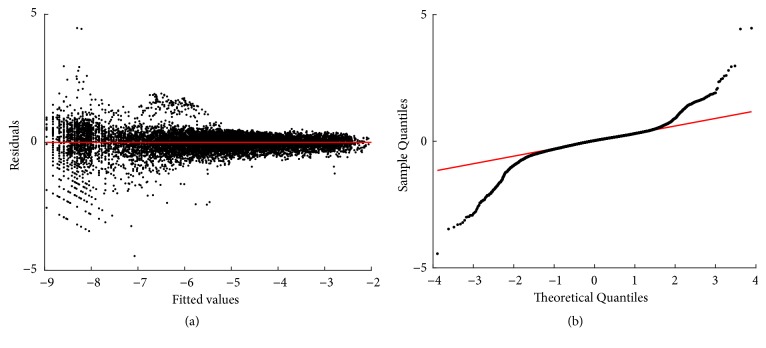
Residuals and normal probability plots ((a) and (b) one to one) for the fitting of the biphasic regression model to processed data. Compared with the TAMA fit, distribution of residuals about the zero line improved. Moreover, the Q-Q plot shows heavier tails than those expected for a normal distribution.

**Figure 14:**
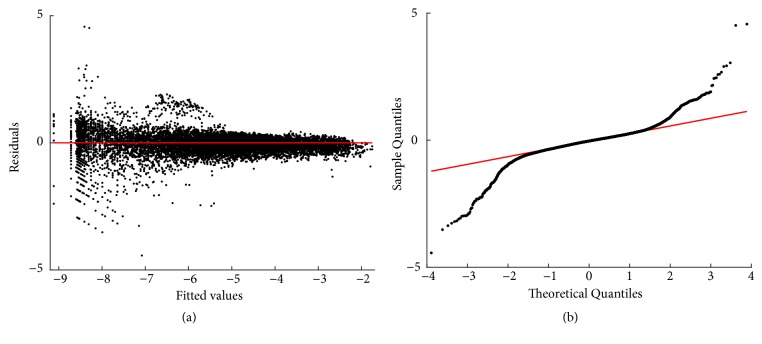
Residuals and normal probability plots ((a) and (b) one to one) for the fitting of the polynomial regression model of (D.8) through (D.10). Compared with TAMA results, distribution of residuals about the zero line improved. Normal Q-Q plot reveals a large plateau, where residuals conform to a normal distribution, but anyhow heavier tails than expected for such a pattern remain.

**Figure 15:**
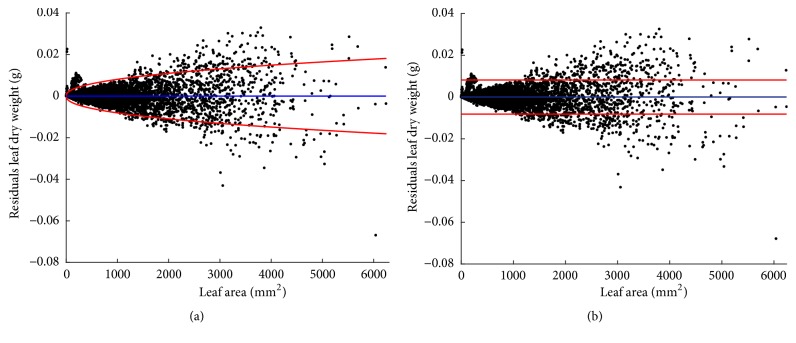
Diagram of dispersion of leaf dry weight residuals compared to leaf area for the fits heteroscedastic and homoscedastic models of (E.1) and (E.5), respectively. Region bounded by red lines determines (95%) confidence intervals for residuals.

**Figure 16:**
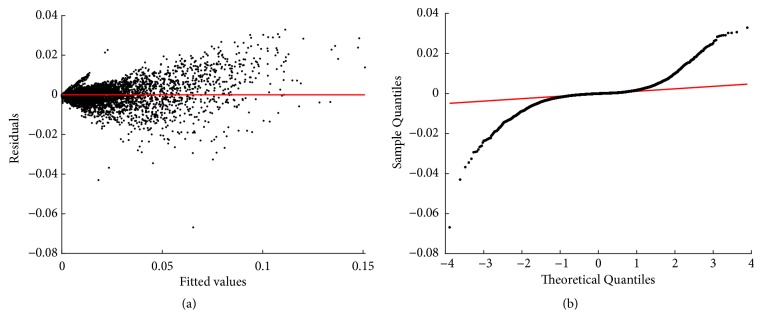
Residuals and normal probability plots ((a) and (b) one to one) for the fitting of the nonlinear heteroscedastic regression model of (E.1). We can observe a slightly biased distribution of residuals around the zero line. Also, normal Q-Q plot in (b) displays heavier tails than those expected for a normal distribution.

**Table 1:** Summary of notation convention of the bivariate scaling model of (1) and its generalization to account for curvature as given by (3). GS stands for geometrical space, AS stands for arithmetical space, and CF means correction factor of retransformation bias.

	Typical bivariate	Eq.	Generalized for curvature	Eq.
Basic form	*w* = *βa*^*α*^	(1)	*w* = *β*(*a*)*a*^*α*(*a*)^	(3)

Regression equation in GS	*v* = ln⁡*β* + *αu* + *ϵ*	(2), (D.1)	*v* = *v*_*C*_(*a*, *u*) + *ϵ*	(4)

Mean response GS	*E*_*T*_(*v* | *u*) = ln⁡*β* + ln⁡*α*	(B.4)	*E*_*C*_(*v* | *u*) = ln⁡*β*(*a*) + *α*(*a*)*u*(*a*)	(B.1)

Back transformation AS	*w* = *e*^*E*_*T*_(*v* | *u*)^*e*^*ϵ*^	(B.5)	*w* = *e*^*E*_*C*_(*v* | *u*)^*e*^*ϵ* ^	(B.2)

Mean response AS	*E*_*Tδ*_(*w* | *a*) = *βa*^*α*^*δ*(*ϵ*) *δ*(*ϵ*) = *E*(*e*^*ϵ*^)	(B.6)	*E*_*Cδ*_(*w* | *a*) = *β*(*a*)*a*^*α*(*a*)^*δ*(*ϵ*) *δ*(*ϵ*) = *E*(*e*^*ϵ*^)	(B.3)

CF Baskerville	*E*_*TB*_(*w* | *a*) = *βa*^*α*^ *δ*_*B*_(*ϵ*) *δ*_*B*_(*ϵ*) = *e*^*σ*^2^/2^	(B.6), (C.1)	*E*_*CB*_(*w* | *a*) = *β*(*a*)*a*^*α*(*a*)^ *δ*_*B*_(*ϵ*)	(B.3), (C.1)

CF Duan	*E*_*TD*_(*w* | *a*) = *βa*^*α*^*δ*_*D*_(*ϵ*) $\delta_{D}\left(\epsilon \right)\;=\;\frac{\sum_{1}^{m}e^{\epsilon_{j}}}{m\;}$	(B.6), (C.2)	*E*_*C*_(*w* | *a*) = *β*(*a*)*a*^*α*(*a*)^*δ*_*D*_(*ϵ*)	(B.3), (C.2)

CF Zeng and Tang	*E*_*TZT*_(*w* | *a*) = *βa*^*α*^*δ*_*ZT*_(*ϵ*) $\delta_{ZT}\left(\epsilon \right)=1+\frac{\sigma^{2}}{2}$	(B.6), (C.3)	*E*_*CZT*_(*w* | *a*) = *β*(*a*)*a*^*α*(*a*)^*δ*_*ZT*_(*ϵ*)	(B.3), (C.3)

CF n-partial sum	*E*_*Tn*_(*w* | *a*) = *βa*^*α*^*δ*_*n*_(*ϵ*) $\delta_{n}\left(\epsilon \right)=\sum_{0}^{n}\frac{E(\epsilon^{k})}{k!}$	(B.6), (C.4)	*E*_*Cn*_(*w* | *a*) = *β*(*a*)*a*^*α*(*a*)^*δ*_*n*_(*ϵ*)	(B.3), (C.4)

**Table 2:** Notation convention for curvature models in geometrical space. We include the biphasic and polynomial characterizations of the generalized allometric model of (3).*GS* stands for geometrical space, *AS* stands for arithmetical space, and *CF* means correction factor of retransformation bias of the regression error.

	Biphasic model	Eq.	Polynomial model	Eq.
Basic form	*w* = *β*_*B*_(*a*)*a*^*α*_*B*_(*a*)^	(B.11)	*w* = *β*_*P*_(*a*)*a*^*α*_*P*_(*a*)^	(B.27)

Regression equation	*v* = *v*_*B*_(*a*, *u*) + *ϵ*	(8), (D.4)	*v* = *v*_*P*_(*a*, *u*) + *ϵ*	(B.32), (D.8)

Mean response GS	*E*_*B*_(*v* | *u*) = ∑_1_^2 ^*ϑ*_*i*_(*u*(*a*))*f*_*i*_(*u*(*a*))	(B.18)	*E*_*P*_(*v* | *u*) = ∑_0_^*m*^*p*_*k*_*u*(*a*)^*k*^	(B.33)

Back transformation AS	*w* = *β*_*B*_(*a*)*a*^*α*_*B*_(*a*)^*e*^*ϵ*^	(B.21)	*w* = *β*_*P*_(*a*)*a*^*α*_*P*_(*a*)^*e*^*ϵ*^	(B.34)

Mean response AS	*E*_*Bδ*_(*w* | *a*) = *β*_*B*_(*a*)*a*^*α*_*B*_(*a*)^*δ*(*ϵ*) *δ*(*ϵ*) = *E*(*e*^*ϵ*^)	(B.22)	*E*_*P*_(*w* | *a*) = *β*_*P*_(*a*)*a*^*α*_*P*_(*a*)^*δ*(*ϵ*) *δ*(*ϵ*) = *E*(*e*^*ϵ*^)	(B.35)

CF Baskerville	*E*_*BB*_(*w* | *a*) = *β*_*B*_(*a*)*a*^*α*_*B*_(*a*)^ *δ*_*B*_(*ϵ*)	(B.22), (C.1)	*E*_*PB*_(*w* | *a*) = *β*_*P*_(*a*)*a*^*α*_*P*_(*a*)^*δ*_*B*_(*ϵ*)	(B.35), (C.1)

CF Duan	*E*_*BD*_(*w* | *a*) = *β*_*B*_(*a*)*a*^*α*_*B*_(*a*)^*δ*_*D*_(*ϵ*)	(B.22), (C.2)	*E*_*PD*_(*w* | *a*) = *β*_*P*_(*a*)*a*^*α*_*P*_(*a*)^*δ*_*D*_(*ϵ*)	(B.35), (C.2)

CF Zeng and Tang	*E*_*BZT*_(*w* | *a*) = *β*_*B*_(*a*)*a*^*α*_*B*_(*a*)^*δ*_*ZT*_(*ϵ*)	(B.22), (C.3)	*E*_*PZT*_(*w* | *a*) = *β*_*P*_(*a*)*a*^*α*_*P*_(*a*)^*δ*_*ZT*_(*ϵ*)	(B.35), (C.3)

CF n-partial sum	*E*_*Bn*_(*w* | *a*) = *β*_*B*_(*a*)*a*^*α*_*B*_(*a*)^*δ*_*n*_(*ϵ*)	(B.22), (C.4)	*E*_*Pn*_(*w* | *a*) = *β*_*P*_(*a*)*a*^*α*_*P*_(*a*)^*δ*_*n*_(*ϵ*)	(B.35), (C.4)

**Table 3:** Assessment of geometrical space fitted models. Comparison took into account goodness-of-fit statistics, that is, the coefficient of determination (R^2^) standard error of estimate (SEE), mean prediction error (MPE), total relative error (TRE), average systematic error (ASE), and mean percent standard error (MPSE) ([63–65]). Besides, we considered concordance correlation coefficient (*ρ*) [61] and Akaike's information index (AIC) [66].

Method	AIC	*ρ*	R^2^	SEE	TRE	ASE	MPE	MPSE
TAMA	15069.9	0.9505	0.9045	0.5135	-0.0286	-0.2462	-0.1879	5.7877
Biphasic	12578.0	0.9614	0.9256	0.4530	0.0037	0.0286	-0.1658	5.1446
Polynomial	12615.0	0.9614	0.9241	0.4576	0.8497	1.2008	-0.1675	5.3187

**Table 4:** Comparison of reproducibility strength statistic for the biphasic mean response *E*_*Bδ*_(*w* | *a*) as calculated by different forms of the correction factor for bias of retransformation of the regression error **ϵ**. The *ρ*(*δ*(*ϵ*)) symbol stands for the concordance correlation value associated with *δ*(*ϵ*).

CF	*ρ*(*δ*(*ϵ*))	R^2^	SEE	TRE	ASE	MPE	MPSE
*δ*_*D*_(*ϵ*)	0.9664	0.9256	0.0049	-9.2196	4.48e-13	0.8133	31.2839
*δ*_*ZT*_(*ϵ*)	0.9687	0.9319	0.0047	-7.5530	1.8359	0.7780	31.4388
*δ*_*n*_(*ϵ*)	0.9727	0.9464	0.0042	1.9516	12.3058	0.6903	33.9525

**Table 5:** Notation convention for the nonlinear heteroscedastic and homoscedastic models of (14) and (15), respectively.

Nonlinear model	Heteroscedastic	Eq.	Homoscedastic	Eq.
Regression equation	*w* = *β*_*θ*_ *a*^*α*_*θ*_^ + *a*^*θ*^*ϵ* *ϵ* = *N*(0, *a*^2*θ*^*σ*^2^)	(14), (E.1)	*w* = *β*_*o*_ *a*^*α*_*o*_^ + *ϵ* *ϵ* ~ *N*(0, *σ*^2^)	(15), (E.5)
Mean response function	*E*_*θ*_(*w* | *a*) = *β*_*θ*_*a*^*α*_*θ*_^	(B.45)	*E*_*o*_(*w* | *a*) = *β*_*o*_ *a*^*α*_*o*_^	(B.46)

**Table 6:** Assessment of models fitted in arithmetical space. This includes the nonlinear homoscedastic and heteroscedastic protocols.

Method	AIC	*ρ*	R^2^	SEE	TRE	ASE	MPE	MPSE
Heteroscedastic	-92761.13	0.972	0.9467	0.0041	0.0311	26.1073	0.6882	50.2781
Homoscedastic	-81386.51	0.972	0.9467	0.0041	0.2954	28.4527	0.6881	51.7400

**Table 7:** Assessment of models in arithmetical space. This includes the nonlinear heteroscedastic and biphasic protocols.

Method	AIC	*ρ*	R^2^	SEE	TRE	ASE	MPE	MPSE
Heteroscedastic	-92761.13	0.972	0.946	0.004	0.031	26.107	0.690	50.278
Biphasic	-96879.30	0.972	0.946	0.004	1.951	12.305	0.690	33.952

**Table 8:** Comparison of reproducibility strengths of proxies of average leaf biomass in shoots resulting from the biphasic polynomial or nonlinear heteroscedastic protocols.

Method	*ρ*	R^2^	SEE	TRE	ASE	MPE	MPSE
Biphasic	0.997	0.994	8.729e-04	2.408	2.229	3.486	5.080
Polynomial (m=6)	0.996	0.992	0.001	-2.456	-1.033	4.807	5.366
Heteroscedastic	0.997	0.994	7.722e-04	0.983	-0.726	3.084	5.645

**Table 9:** Values of minimum, maximum, sample mean, and quantiles for measurements of eelgrass dry weight (*w*[gr]) and area (*a*[mm^2^]) (and their logarithms) of a sample of 10412 leaves before applying a data-cleaning procedure aimed to eliminate outliers.

	Min	0.10	0.25	0.50	Mean	0.75	0.90	Max
*w*[gr]	0.00001	0.00042	0.00154	0.00564	0.01293	0.01477	0.035443	0.38058
*a*[mm^2^]	2.00	32.50	127.5	355.2	690.5	836.0	1859.00	7868.0
ln⁡*w*	-11.513	-7.7753	-6.4760	-5.1779	-5.4001	-4.2152	-3.33983	-0.9661
ln⁡*a*	0.6931	3.48124	4.8481	5.8728	5.6729	6.7286	7.527794	8.9706

**Table 10:** Values of minimum, maximum, sample mean, and quantiles for measurements of eelgrass dry weight (*w*[gr]) and area (*a*[mm^2^]) (and their logarithms) of a sample of 10023 leaves remaining after applying a data-cleaning procedure aimed to eliminate outliers.

	Min	0.10	0.25	0.50	Mean	0.75	0.90	Max
*w*[gr]	0.00001	0.00041	0.00144	0.00529	0.01211	0.01373	0.03381	0.15096
*a*[mm^2^]	2.00	30.00	124.00	352.50	672.83	828.00	1835.80	6240.00
ln⁡*w*	-11.5129	-7.7994	-6.5431	-5.2419	-5.4593	-4.2878	-3.3868	-1.8907
ln⁡*a*	.6931	3.4012	4.8203	5.8651	5.6518	6.7190	7.5152	8.7387

**Table 11:** Parameter estimates of straight lines fitted on raw (10412) and processed data (10023) and shown in the Q-Q plots of Figure 8.

	Estimate	Standard Error
Raw data		
Intercept	141.22	3.88
slope	42493.46	163.29

Processed data		
Intercept	111.20	2.50
Slope	46360.00	114.90

**Table 12:** Fitting results of regression model of the TAMA protocol (cf. (D.1) through (D.3)). CI stands for confidence interval, RSE stands for standard error of residuals, RMS means multiple R-squared, and ARS is adjusted R-squared.

Statistics of residuals					
*Minimum*	*1Q*	*Median*	*3Q*		*Maximum*
-4.7088	-0.2344	0.0281	0.2244		4.7169

*Parameters*	*Estimate*	*Std. Error*	*t value*	*Pr*(>|*t*|)	*C.I. (95*%)
*α*	1.028222	0.003335	308.27	<2e-16	(1.021684, 1.03476)
ln⁡*β*	-11.270569	0.019534	-576.94	<2e-16	(-11.308861, -11.23228)

*RSE σ*		0.5131 on 10021 df			
*MRS*		0.9046			
*ARS*		0.9046			
*F-statistic*		9.504e+04 on 1 and 10021 df			
*p-value*		< 2.2e-16			

**Table 13:** Fitting results of the biphasic regression model of (D.4) through (D.7) to processed data. CI stands for confidence interval and RSE for standard error of residuals.

Residual statistics					
*Minimum*	*1Q*		*Median*	*3Q*	*Maximum*
-4.4406	-0.1948		0.0240	0.2072	4.4626

*Parameters*	*Estimate*	*Std. Error*	*t value*	*Pr(>*|*t*|)	*CI (95*%)
ln⁡*β*_1_	-9.190321	0.052812	-174.0	<2e-16	(-9.2938, -9.0868)
ln⁡*β*_2_	-11.9381	0.093895	-127.1	<2e-16	(-12.1221, -11.7541)
*u*_*b*_	3.550709	0.032744	108.4	<2e-16	(3.4865, 3.6149)
*α*_1_	0.336339	0.020042	16.8	<2e-16	(0.2971, 0.3756)
*α*_2_	1.164558	0.004248	274.1	<2e-16	(1.1562, 1.1729)

*RSE σ*		0.452947 on 10018 df			

**Table 14:** Fitting results of the polynomial regression model. CI stands for confidence interval, RSE stands for standard error of residuals, RMS means multiple R-squared, and ARS is adjusted R-squared.

Residual statistics					
*Minimum*	*1Q*		*Median*	*3Q*	*Maximum*
-4.4207	-0.1947		0.0248	0.2072	4.5703

* Parameters*	*Estimate*	*Std. Error*	*t value*	*Pr(>*|*t*|)	*CI (95*%)
*p*_0_	-11.080	0.4136	-26.783	<2e-16	(-11.889e+1, -10.267e+1)
*p*_1_	4.602	0.7411	6.209	5.54e-10	(3.1489, 6.0543)
*p*_2_	-3.2120	0.5027	-6.390	1.74e-10	(-4.1976, -2.2267)
*p*_3_	1.0620	0.1671	6.355	2.17e-10	(0.7344, 1.3894)
*p*_4_	-0.1678	0.02911	-5.765	8.43e-09	(-0.22483, -0.11072)
*p*_5_	0.01287	0.002549	5.047	4.57e-07	(0.0078689, 0.017863)
*p*_6_	- 3.854e-04	8.854e-05	-4.353	1.36e-05	(-0.00055897, -0.00021186)

*RSE σ*		0.4538 on 10016 df			
*MRS*		0.9254			
*ARS*		0.9254			
*F-statistic*		2.071e+4 on 6 and 10016 df			
*p-value*		< 2.2e-16			

**Table 15:** Maximum likelihood estimates of parameters for the heteroscedastic and homoscedastic regression models ((E.1) and (E.5) one to one). CI stands for confidence interval.

Heteroscedastic fit			

*Parameter*	*Estimate*	*Std. Error*	*CI (95*%)
*α*_*θ*_	1.1298	0.003414	(1.1230, 1.1366)
*β*_*θ*_	7.073e-06	1.804e-07	(6.7123e-06, 7.434e-06)
*θ*	0.4415	0.003378	(0.4347, 0.4483)
*σ*	1.951e-04	3.972e-06	(1.8716e-04, 2.0304e-04)

Homoscedastic fit			

*Parameter*	*Estimate*	*Std. Error*	*CI (95*%)
*α*_*o*_	1.1365	0.003560	(1.1293, 1.1437)
*β*_*o*_	6.724e-06	1.883e-07	(6.347e-06, 7.10e-06)
*σ*	0.004173	2.948e-05	(0.00411, 0.004229

## Data Availability

Data will be provided by the corresponding author upon usage agreement.

## Appendix

### A. Data Exploratory Analysis

This appendix presents an exploratory analysis of raw and processed data sets contemplated in this examination.

Table 9 describes the distribution pattern, in terms of quantiles, for a sample of 10412 measurements of eelgrass leaf weights and related areas taken over 13 months conforming the present raw data. The first four columns in the uppermost row label the minimum followed by quantiles of probability 0.1, 0.25, and 0.50. Correspondingly, the fifth column in the first row presents the sample mean followed by quantiles of probability 0.75 and 0.90 before the maximum. Second row presents leaf dry weight values (*w*) and third row shows corresponding areas *a*. Third and fourth rows present transformed ln⁡*w* and ln⁡*a* values one to one. Similarly, Table 10 shows the variation pattern of the 10023 observations resulting after applying to raw data a mean plus or minus two standard deviations outlier's removal procedure [58] designed to remove replicates considered significantly discrepant from the mean response function of a simple allometric model for a leaf dry weight response in terms of a leaf area covariate.

Comparing quantile values for the leaf dry weight (*w*) and corresponding area (*a*) variables reported in Tables 9 and 10 for crude and processed data respectively, we can ascertain that in spite of removing a large number of discrepant observations by data cleaning, both original and remnant sets show an equivalent distribution pattern. This similarity in distribution before and after data processing of the data can be better perceived in the Q-Q (quantile-quantile) graphs observed in Figure 8 for raw (a) and processed data (b), respectively.

Q-Q plots in Figure 8 compare the distributions (regardless what they are) of the leaf area *a* and associated dry weight *w* variables. The linear relationship observed between the quantiles of both variables underlines that both variables have similar distributions, although with different parameters. This similarity between distributions of both variables is observed for both sets of observations. The great similarity of the fitted straight lines for both sets of observations in Table 11 confirms that the referred distribution pattern does not change after data processing.

Figures 9 and 10 display boxplots for the 13 months' long sampling scheme. We can learn that, for raw data, from month 2 to month 6, a reduction in the values of both leaf dry weight and linked area occurred. This is perhaps explained by an increase in temperature during those months. Processed data exhibits similar dynamics through time for these variables. Moreover, the overall variation patterns of raw and processed data, throughout the 13 months of sampling, are similar

### B. Derivation of Regression Protocols

In this appendix, we first present notation convention for statistics related to the generalized bivariate model of (3). We then explain how the different regression protocols addressed in this examination derive from this paradigm. We also include notation convention for related statics.

#### B.1. Generalized Bivariate Allometric Model

A transformation *v* = ln⁡*w* and *u*(*a*) = ln⁡*a* of (3) establishes the generalized regression model in geometrical space given by (4). The term *v*_*C*_(*a*, *u*) stands for the corresponding mean response function. Using the customary notation convention, we represent *v*_*C*_(*a*, *u*) through symbol *E*_*C*_(*v*∣*u*); that is,

(B.1) $$\begin{array}{c} E_{C}\left(\;v\;|\;u\;\right)=\ln\beta \left(\;a\;\right)+\alpha \left(\;a\;\right)u\left(\;a\;\right). \end{array}$$

Furthermore, back-transformation *w* = *e*^*v*^ of (4) leads to the result

(B.2) $$\begin{array}{c} w=e^{E_{C}\left(v\;|\;u\right)}e^{\epsilon }. \end{array}$$

Thus, *e*^*ϵ*^ is understood as a multiplicative error term. Denoting by the symbol *E*_*Cδ*_(*w*∣*a*) the corresponding mean response function in arithmetical space, from (B.1) and (B.2), we have

(B.3) $$\begin{array}{c} E_{C\delta }\left(\;w\;|\;a\;\right)\left(\;w\;|\;a\;\right)=\beta \left(\;a\;\right)a^{\alpha \left(a\right)}\delta \left(\;\epsilon \;\right) \end{array}$$

with *δ*(*ϵ*) = *E*(*e*^*ϵ*^) interpreted as a correction factor of bias of retransformation of the regression error *ϵ*.

#### B.2. Traditional Analysis Method of Allometry (TAMA)

The bivariate allometric model of (1) derives from the model of (3) setting the scaling parameters constant; that is, *β*(*a*) = *β* and *α*(*a*) = *α*. The resultant regression model in geometrical space is given by (2). Corresponding mean response function will be denoted here by means of the symbol *E*_*T*_(*v*∣*u*). Hence, from (2) we have

(B.4) $$\begin{array}{c} E_{T}\left(\;v\;|\;u\;\right)=\ln\beta +\alpha u. \end{array}$$

Retransformation *w* = *e*^*v*^ of (2) leads to the result

(B.5) $$\begin{array}{c} w=e^{E_{T}\left(v\;|\;u\right)}e^{\epsilon }. \end{array}$$

And linked mean response function in arithmetical space denoted through *E*_*Tδ*_(*w*∣*a*) becomes

(B.6) $$\begin{array}{c} E_{T\delta }\left(\;w\;|\;a\;\right)={\beta a}^{\alpha }\delta \left(\;\epsilon \;\right) \end{array}$$

#### B.3. Biphasic Model in Geometrical Space

Here, we explain the result of (8), as well as the notation convention for related statistics. In order to characterize a biphasic form of the model of (3), we introduce fixed values *a*_*min*_ and *a*_*max*_, so that the covariate *a* takes values in a range *a*_*min*_ ≤ *a* ≤ *a*_*max*_. We then conceive *a*_*b*_ as a fixed value of *a* satisfying *a*_*min*_ ≤ *a*_*b*_ ≤ *a*_*max*_. And recalling the transformation *v* = ln⁡*w* and *u*(*a*) = ln⁡*a*, we take *u*_*b*_ = ln⁡*a*_*b*_ as a breaking point for transition between two different phases of the variation of *v*. Moreover, in the model of (3), we let *β*(*a*) = *β*_*B*_(*a*) and *α*(*a*) = *α*_*B*_(*a*) given by

(B.7) αBa=∑12ϑiuaαi

(B.8) βBa=exp⁡∑12ϑiualn⁡βi,

(B.9) ϑiua=2−i1−Hu−ubfor  i=1i−1Hu−ubfor  i=2

where *H*(*x*) is the Heaviside [60] function defined through

(B.10) $$\begin{array}{c} H\left(\;x\;\right)=\left\{\begin{array}{cc} 0 & x<0\\ 1 & x\geq 0. \end{array}\right. \end{array}$$

Then, the biphasic form of (3) is formally represented by

(B.11) $$\begin{array}{c} w=\beta_{B}\left(\;a\;\right)a^{\alpha_{B}\left(a\right)}. \end{array}$$

Denoting by *v*_*B*_(*a*, *u*) the resulting form of *v*_*C*_(*a*, *u*), (5) yields

(B.12) $$\begin{array}{c} v_{B}\left(\;a,u\;\right)=\ln\beta_{B}\left(\;a\;\right)+\alpha_{B}\left(\;a\;\right)u\left(\;a\;\right). \end{array}$$

Then, (B.7), (B.8), and (B.12) imply that

(B.13) $$\begin{array}{c} v_{B}\left(\;a,u\;\right)=\overset{2}{\underset{1}{\sum }}\vartheta_{i}\left(\;u\left(\;a\;\right)\;\right)f_{i}\left(\;u\left(\;a\;\right)\;\right), \end{array}$$

where

(B.14) $$\begin{array}{c} f_{i}\left(\;u\left(\;a\;\right)\;\right)=\ln\beta_{i}+\alpha_{i}u\left(\;a\;\right). \end{array}$$

This explains (8). Since *w* as given by (3) is assumed to be a continuous function of *a*, this property ought to be satisfied by the biphasic model; that is, we require to set a condition, *f*_1_(*u*_*b*_) = *f*_2_(*u*_*b*_). This leads to the equation

(B.15) $$\begin{array}{c} \ln\frac{\beta_{1}}{\beta_{2}}=\left(\;\alpha_{2}-\alpha_{1}\;\right)u_{b}. \end{array}$$

Moreover, *v*_*B*_(*a*, *u*) as given by (8) can be equivalently represented by

(B.16) $$\begin{array}{c} v_{B}\left(\;a,u\;\right)=\left\{\begin{array}{cc} \ln\beta_{1}+\alpha_{1}u & u\leq u_{b}\\ \ln\beta_{2}+\alpha_{2}u & u>u_{b}, \end{array}\right. \end{array}$$

with the continuity condition of (B.15).

Correspondingly, the regression model of (4) in its biphasic form becomes

(B.17) $$\begin{array}{c} v=v_{B}\left(\;a,u\;\right)+\epsilon . \end{array}$$

As we explained in (4), *ϵ* is a residual error term conceived as a random variable distributed according to an unknown distribution *ψ* having mean *μ* and variance set by a function *σ*^2^(*u*) of the covariate *u*; that is, *ϵ* ~ *ψ*(*μ*, *σ*^2^(*u*)).

The form of the generalized mean response *E*_*C*_(*v*∣*u*) for the biphasic model is denoted through *E*_*B*_(*v*∣*u*) and becomes

(B.18) $$\begin{array}{c} E_{B}\left(\;v\;|\;u\;\right)=\overset{2}{\underset{1}{\sum }}\vartheta_{i}\left(\;u\left(\;a\;\right)\;\right)f_{i}\left(\;u\left(\;a\;\right)\;\right). \end{array}$$

It follows from (B.16) that the expression for *E*_*B*_(*v*∣*u*) can be equivalently represented by

(B.19) $$\begin{array}{c} E_{B}\left(\;v\;|\;u\;\right)=\left\{\begin{array}{cc} \ln\beta_{1}+\alpha_{1}u & u\leq u_{b}\\ \ln\beta_{2}+\alpha_{2}u & u>u_{b}. \end{array}\right. \end{array}$$

Back-transformation *w* = *e*^*v*^ of (B.17) leads to the result

(B.20) $$\begin{array}{c} w=e^{E_{B}\left(v\;|\;u\right)}e^{\epsilon }, \end{array}$$

which can be written in the form

(B.21) $$\begin{array}{c} w=\beta_{B}\left(\;a\;\right)a^{\alpha_{B}\left(a\right)}e^{\epsilon }. \end{array}$$

Then, the mean response function in arithmetical space *E*(*w*∣*a*) becomes

(B.22) $$\begin{array}{c} E_{B\delta }\left(\;w\;|\;a\;\right)=\beta_{b}\left(\;a\;\right)a^{\alpha_{b}\left(a\right)}\delta \left(\;\epsilon \;\right). \end{array}$$

#### B.4. Polynomial Model in Geometrical Space

In this section, we explain how (12) can be derived from the generalized structure of model of (3). We also set the notation convention for related statistics. For these aims, we begin by considering polynomials *ϕ*_*β*_(*a*) and *ϕ*_*α*_(*a*) given by

(B.23) ϕβa=∑0nβkuak

(B.24) ϕαa=∑0nαkuak,

where, for 1 ≤ *k* ≤ *m*, *α*_*k*_ and *β*_*k*_ stand for coefficients. Now, let *α*(*a*) = *α*_*P*_(*a*) and *β*(*a*) = *β*_*P*_(*a*), where

(B.25) αPa=ϕαa

(B.26) βPa=eϕβa.

This way, we obtain a representation of the model of (3) in the form

(B.27) $$\begin{array}{c} w=\beta_{P}\left(\;a\;\right)a^{\alpha_{P}\left(a\right)}. \end{array}$$

Now, denoting by means of *v*_*P*_(*a*, *u*) the associated form of *v*_*C*_(*a*, *u*), according to (5), we have

(B.28) $$\begin{array}{c} v_{P}\left(\;a,u\;\right)=\ln\beta_{P}\left(\;a\;\right)+\alpha_{P}\left(\;a\;\right)u\left(\;a\;\right). \end{array}$$

From (B.23) through (B.28), we obtain

(B.29) $$\begin{array}{c} v_{P}\left(\;a,u\;\right)=\overset{n}{\underset{0}{\sum }}\beta_{k}u{\left(\;a\;\right)}^{k}+\overset{n}{\underset{0}{\sum }}\alpha_{k}u{\left(\;a\;\right)}^{k+1}. \end{array}$$

Rearranging, we ascertain that *v*_*B*_(*u*, *a*) takes on the polynomial form

(B.30) $$\begin{array}{c} v_{P}\left(\;a,u\;\right)=\overset{m}{\underset{0}{\sum }}p_{k}u{\left(\;a\;\right)}^{k} \end{array}$$

where *m* = *n* + 1, and

(B.31) p0=β0,pk=βk+αk−1for  1≤k≤m−1,pm=αm.

This explains the result of (12). Correspondingly, a polynomial characterization of the regression model of (4) takes the form

(B.32) $$\begin{array}{c} v=v_{P}\left(\;a,u\;\right)+\epsilon , \end{array}$$

with *ϵ* being a residual error as described in (4). The form of the generalized mean response *E*_*c*_(*v*∣*u*) for the polynomial characterization is denoted through *E*_*P*_(*v*∣*u*). It becomes

(B.33) $$\begin{array}{c} E_{P}\left(\;v\;|\;u\;\right)=\overset{m}{\underset{0}{\sum }}p_{k}u{\left(\;a\;\right)}^{k}. \end{array}$$

Back-transformation *w* = *e*^*v*^ of (B.32) leads to

(B.34) $$\begin{array}{c} w=e^{E_{P}\left(v\;|\;u\right)}e^{\epsilon }. \end{array}$$

And the mean response function in arithmetical space *E*_*Pδ*_(*w*∣*a*) becomes

(B.35) $$\begin{array}{c} E_{P\delta }\left(\;w\;|\;a\;\right)=\beta_{P}\left(\;a\;\right)a^{\alpha_{P}\left(a\right)}\delta \left(\;\epsilon \;\right) \end{array}$$

#### B.5. Nonlinear Regression Models

In this section, we explain how the nonlinear heteroscedastic model of (14) can be also associated with (3). We also explain the notation convention for related statistics. For these aims, we begin by noticing that *v*_*C*_(*a*, *u*) in (5) can be also written in the form

(B.36) $$\begin{array}{c} v_{C}\left(\;a,u\;\right)=\ln\beta +\alpha u+\lambda \left(\;a\;\right) \end{array}$$

where *u* = ln⁡*a* and

(B.37) $$\begin{array}{c} \lambda \left(\;a\;\right)=\ln\left(\;\frac{\beta \left(a\right)}{\beta }\;\right)+\left(\;\alpha \left(\;a\;\right)-\alpha \;\right)\ln a. \end{array}$$

Now, if *β*(*a*) and *α*(*a*) are as defined in (3), then (B.36) turns out to be nonlinear in geometrical space. Moreover, since from (B.36) we have

(B.38) $$\begin{array}{c} e^{v_{C}\left(a,u\right)}={\beta a}^{\alpha }e^{\lambda \left(a\right)} \end{array}$$

noticing that, from (4), *e*^*v*_*C*_(*a*, *u*)^ = *β*(*a*)*a*^*α*(*a*)^, then solving for *e*^*λ*(*a*)^ above yields

(B.39) $$\begin{array}{c} e^{\lambda \left(a\right)}=\frac{\beta \left(a\right)a^{\alpha \left(a\right)}}{{\beta a}^{\alpha }}. \end{array}$$

Then, we have that the generalized curvature model of (3) can be also written in the form

(B.40) $$\begin{array}{c} w={\beta a}^{\alpha }e^{\lambda \left(a\right)} \end{array}$$

Thus, defining

(B.41) $$\begin{array}{c} \varepsilon \left(\;a\;\right)=\beta a^{\alpha -\theta }\left(\;e^{\lambda \left(a\right)}-1\;\right), \end{array}$$

(B.39) implies

(B.42) $$\begin{array}{c} w={\beta_{\theta }a}^{\alpha_{\theta }}+a^{\theta }\varepsilon \left(\;a\;\right), \end{array}$$

which suggests the nonlinear heteroscedastic regression model of (14). That is,

(B.43) $$\begin{array}{c} w={\beta_{\theta }a}^{\alpha_{\theta }}+a^{\theta }\epsilon , \end{array}$$

which can be considered as a tool to analyze curvature in geometrical space. Particularly, by setting *θ* = 0, we can consider the homoscedastic model

(B.44) $$\begin{array}{c} w={\beta_{0}a}^{\alpha_{0}}+\epsilon , \end{array}$$

where in (B.43) and (B.44) the error term *ϵ* is assumed to be normally distributed random variable having zero mean and homogeneous variance; that is, *ϵ* ~ *N*(0, *σ*^2^).

It turns out that the mean response function *E*_*θ*_(*w*∣*a*) associated with the heteroscedastic model becomes

(B.45) $$\begin{array}{c} E_{\theta }\left(\;w\;|\;a\;\right)={\beta_{\theta }a}^{\alpha_{\theta }}. \end{array}$$

Similarly, the mean response function *E*_0_(*w*∣*a*) associated with the homoscedastic model becomes

(B.46) $$\begin{array}{c} E_{0}\left(\;w\;|\;a\;\right)=\beta_{0}a^{\alpha_{0}}. \end{array}$$

Finally, by setting *α*(*a*) = *α* and *β*(*a*) = *β* + *c*/*a*^*α*^ in (3), a log transformation *v* = ln⁡*w* and *u* = ln⁡*a* leads to the nonlinear regression model in geometrical space:

(B.47) $$\begin{array}{c} v=\ln\left(\;\beta +ce^{-\alpha u}\;\right)+\alpha u+\epsilon \end{array}$$

with the error term assumed to be normally distributed with zero mean and constant variance *σ*^2^. Then, back-transformation *w* = *e*^*v*^ of (B.47) to arithmetical space yields

(B.48) $$\begin{array}{c} w=\left(\;\beta a^{\alpha }+c\;\right)e^{\epsilon }. \end{array}$$

The mean response function in arithmetical space becomes

(B.49) $$\begin{array}{c} E\left(\;w\;|\;a\;\right)=\left(\;\beta a^{\alpha }+c\;\right)\delta \left(\;\epsilon \;\right) \end{array}$$

Packard [59] asserts that, commonly, any data set on arithmetical scale that is consistently described by a three-parameter power function will track a curved path when transformed to the logarithmic scale. Equations (B.45) through (B.47) provide a formal set-up accommodating such a statement.

### C. Forms of the Correction Factor of Bias of Retransformation of the Regression Error

In this appendix, we explain the different characterizations of *δ*(*ϵ*), defined as a correction factor for bias of retransformation of the regression error *ϵ* introduced by (B.3) for *E*_*Cδ*_(*w*∣*a*).

If *ϵ* is normally distributed with zero mean and constant variance *σ*^2^, that is *ϵ* ~ *N*(0, *σ*^2^), resulting form of *δ*(*ϵ*), denoted here by means of the symbol *δ*_*B*_(*ϵ*), is given by

(C.1) $$\begin{array}{c} \delta_{B}\left(\;\epsilon \;\right)=e^{\sigma^{2}/2}. \end{array}$$

*δ*_*B*_(*ϵ*) will be referred to as the Baskerville [51] form of *δ*(*ϵ*).

In case of a nonnormally distributed residual error term *ϵ*, Newman [52] asserts that *δ*(*ϵ*) must take the form provided by Duan's smearing estimate of bias [54]. Here, this form is correspondingly represented by means of the symbol *δ*_*D*_(*ϵ*), and it is calculated by means of

(C.2) $$\begin{array}{c} \delta_{D}\left(\;\epsilon \;\right)=\frac{\sum_{1}^{m}e^{\epsilon_{j}}}{m}, \end{array}$$

with *ϵ*_*j*_ standing for the *j* − *th* residual of the contemplated regression model. Nonetheless, this form of *δ*(*ϵ*) could produce bias overcompensation ([53, 55]). Moreover, *δ*_*D*_(*ϵ*) corresponds to the sample mean of retransformed residuals. Therefore, whenever outliers occur, the actual central tendency of retransformed residuals data could not be represented by *δ*_*D*_(*ϵ*). Under such a circumstance, a suitable form of the correction factor *δ*(*ϵ*) seems indefinite. Moreover, Zeng and Tang [57] suggest a distribution-free form of *δ*(*ϵ*) represented here by means of *δ*_*ZT*_(*ϵ*) and given by

(C.3) $$\begin{array}{c} \delta_{ZT}\left(\;\epsilon \;\right)=1+\frac{\sigma^{2}}{2}. \end{array}$$

We notice that *δ*_*ZT*_(*ϵ*) is actually an approximation to *δ*(*ϵ*) as it corresponds to a three terms' partial sum of the power series expression of *δ*(*ϵ*) assuming *E*(*ϵ*) = 0. By the same token, we can consider an alternative approximation for *δ*(*ϵ*); this is represented through the symbol *δ*_*n*_(*ϵ*) and given by an *n*-terms partial sum of the series representation of *E*(*e*^*ϵ*^); that is,

(C.4) $$\begin{array}{c} \delta_{n}\left(\;\epsilon \;\right)=\overset{n}{\underset{0}{\sum }}\frac{E\left(\epsilon^{k}\right)}{k!}. \end{array}$$

The value of *n* leading to the highest concordance correlation coefficient value between *E*_*Cδ*_(*w*∣*a*) projections and observed values sets the explicit form of *δ*_*n*_(*ϵ*) in (C.4).

### D. Fitting Results of Regresion Models in Geometrical Space

This appendix presents fitting results of the geometrical space protocols derived from the generalized model of (3). This includes TAMA and biphasic and polynomial schemes.

#### D.1. Fitting Results of the TAMA Protocol

Since we are dealing with bivariate data (*w*_*i*_, *a*_*i*_), in arithmetical scale, according to the TAMA protocol, we have to consider pairs (*v*_*i*_, *u*_*i*_), where *v*_*i*_ = ln⁡*w*_*i*_ and *u*_*i*_ = ln⁡*a*_*i*_ for *i* = 1,2,….*n*. Moreover, the linear regression model of (2) becomes

(D.1) $$\begin{array}{c} v_{i}=\ln\beta +\alpha u_{i}+\varepsilon_{i}, \end{array}$$

where the error term *ε*_*i*_ is assumed to be normally distributed with zero mean and constant variance *σ*^2^. Hence, the response *v*_*i*_ has normal distribution *N*(*μ*_*i*_, *σ*^2^) with constant variance *σ*^2^ and a mean *μ*_*i*_(*α*, *β*, *u*_*i*_) expressed as a function of the covariate *u*_*i*_; namely,

(D.2) $$\begin{array}{c} \mu_{i}\left(\;\alpha ,\beta ,u_{i}\;\right)=\ln\beta +\alpha u_{i}. \end{array}$$

Identification method contemplated here relies on finding parameter estimates that maximize the log-likelihood function *l*(*β*, *α*, *σ*). This is given by

(D.3) lβ,α,σ=−n2log⁡2π−∑1nlog⁡σ−12∑1nvi−ln⁡β−αuiσ2.

Fitting results of the linear model of (D.1) through (D.3) to raw data appear in Table 12.

Figure 11(a) displays a biased distribution of residuals around the zero line. The normal Q-Q plot in Figure 11(b) can be considered as a visual test of goodness of fit. In this case, this provides primary evidence against normality of residuals. Indeed, we can learn of a heavy-tails pattern in this plot. Moreover, an Anderson-Darling [67] goodness-of-fit test resulted in a test statistic of 246.7 and in a p value of <2.2e-16, which confirms lack of normality of residuals.

Figure 12 shows spread about TAMA's mean response *E*_*Tδ*_(*w*∣*a*) as produced by different forms of the correction factor *δ*(*ϵ*). Plot suggests moderate bias about *E*_*Tδ*_(*w*∣*a*) for leaves of small-to-medium-sized leaves. Nevertheless, deviations between projected and processed data are notorious for larger area values. Thus, spread in Figure 12 suggests that the TAMA protocol is unsuited for the analysis of present data.

#### D.2. Fitting Results of the Biphasic Model

As before, for data pairs (*v*_*i*_, *u*_*i*_), with *v*_*i*_ = ln⁡*w*_*i*_ and *u*_*i*_ = ln⁡*a*_*i*_, for *i* = 1,2,….*n*, (B.13) and (B.17) yield the regression model

(D.4) vi=ϑ1uiln⁡β1+ϑ2uiln⁡β2+ϑ1uiα1+ϑ2uiα2ui+εi,

with the additive errors *ε*_1_, *ε*_2_,…, *ε*_*n*_ assumed to be normally distributed with zero mean and constant variance *σ*^2^. Moreover, the response *v*_*i*_ has normal distribution *N*(*μ*_*i*_, *σ*^2^) with constant variance *σ*^2^ and a mean *μ*_*i*_(*α*, *β*, *u*_*i*_) expressed as a function of the covariate *u*_*i*_; namely,

(D.5) $$\begin{array}{c} \vartheta_{k}\left(\;u_{i}\;\right)=\left\{\begin{array}{cc} \left(2-k\right)\left(1-H\left(u_{i}-u_{b}\right)\right) & k=1\\ \left(k-1\right)H\left(u_{i}-u_{b}\right) & k=2, \end{array}\right. \end{array}$$

and, hence, the response *v*_*i*_ as given by (D.4) is normally distributed having mean *μ*_*i*_(*u*_*i*_, *π*) with *π* standing for the parameter vector (*β*_1_, *β*_2_, *α*_1_, *α*_2_, *u*_*b*_) and given by

(D.6) μiui,π=ln⁡β1+Hui−ubln⁡β2β1+α1+Hui−ubα2−α1ui

and variance *σ*^2^. Therefore, the associated log-likelihood function *l*(*β*_1_, *β*_2_, *α*_1_, *α*_2_, *u*_*b*_, *σ*) becomes

(D.7) lβ1,β2,α1,α2,ub,σ=−n2log⁡2π−∑1nlog⁡σ−12∑1nwi−μiui,β1,β2,α1,α2,ubσ2.

Table 13 Presents fitting results of the biphasic regression model of (D.4) through (D.7). It is worth mentioning that in this case we are performing a nonlinear fit in geometrical space.

Comparing the behavior of the residuals of the TAMA and biphasic models from Figure 13, we can learn of a relative stabilization of variability of the residues. Nevertheless, the biphasic normal Q-Q plot still shows heavier tails than those expected for a normal distribution. This amounts to primary evidence against normality of the residuals. An Anderson-Darling goodness-of-fit test [67] that resulted in a test statistic of 254.5 and a p value <2.2e-16 confirms a lack of normality for the involved residuals. Nevertheless, compared with the TAMA fit, the normal Q-Q plot for the biphasic model reveals a larger region where data track a normal distribution pattern.

#### D.3. Fitting Results of the Polynomial Model

We now explain the statistical analysis involved in the identification of the polynomial model of (B.27). Again, for data pairs (*v*_*i*_, *u*_*i*_), with *v*_*i*_ = ln⁡*w*_*i*_ and *u*_*i*_ = ln⁡*a*_*i*_, for *i* = 1,2,….*n*, from (B.29) and (B.30), we get the regression model

(D.8) $$\begin{array}{c} v_{i}=\overset{m}{\underset{0}{\sum }}{p_{k}u_{i}}^{k}+\varepsilon_{i}, \end{array}$$

where the additive errors *ε*_1_, *ε*_2_,…, *ε*_*n*_ are independent and identically distributed, with distribution *N*(0, *σ*^2^). Hence, the response *v*_*i*_ above is normally distributed having mean *μ*_*i*_(*u*_*i*_, *π*) with *π* standing for the parameter vector (*p*_0_, *p*_1_,…, *p*_*m*_); namely,

(D.9) $$\begin{array}{c} \mu_{i}\left(\;u_{i},\pi \;\right)=\overset{m}{\underset{0}{\sum }}p_{k}{u_{i}}^{k}. \end{array}$$

Therefore, the associated log-likelihood function *l*(*p*_0_, *p*_1_,…, *p*_*m*_, *σ*) becomes

(D.10) lp0,p1,…pm,σ=−n2log⁡2π−∑1nlog⁡σ−12∑1nwi−μiui,πσ2.

Fitting results of the polynomial regression model of (D.8) through (D.10) are shown in Table 14.

Reviewing fitting results of the polynomial model, we observe similar results to those we found earlier for the fit of the biphasic model. Moreover, Figure 14 displays similar patterns to those shown in Figure 13. Also for this fit, an Anderson-Darling normality test [67] delivered a test statistic 257.1 and a p value <2.2e-16 that yields remarkable evidence against normality of residuals. Again, comparing with TAMA results, distribution of residuals about the zero line improved. Moreover, the Q-Q plot shows a larger plateau, where residuals agree to a normal distribution pattern.

### E. Fitting Results of Direct Nonlinear Regression Models

This appendix presents the exploration of curvature by means of direct nonlinear regression methods. For that aim, we present fitting results of the nonlinear heteroscedastic model of (14). For comparison, we include fitting results of the homoscedastic case of (15).

Since we deal with data pairs (*w*_*i*_, *a*_*i*_) for the heteroscedastic case, the regression equation to be considered turns out to be

(E.1) $$\begin{array}{c} w_{i}=\beta_{\theta }{a_{i}}^{\alpha_{\theta }}+{a_{i}}^{\theta }\epsilon_{i}, \end{array}$$

where *ϵ*_*i*_ is a normally distributed zero mean random variable with standard deviation *σ*. Then the response *w*_*i*_ is normally distributed having mean function *μ*_*i*_(*a*_*i*,_*α*_*θ*_, *β*_*θ*_) given by

(E.2) $$\begin{array}{c} \mu_{i}\left(\;a_{i,}\alpha_{\theta },\beta_{\theta }\;\right)=\beta_{\theta }{a_{i}}^{\alpha_{\theta }}. \end{array}$$

And a variance set as a function *σ*_*i*_^2^(*a*_*i*_) of covariate *a*_*i*_ is, namely,

(E.3) $$\begin{array}{c} \sigma_{i}^{2}\left(\;a_{i}\;\right)=\sigma^{2}a_{i}^{2\theta }. \end{array}$$

Again, fitting this regression model relied on a likelihood approach, that is, obtaining estimates for the parameters *α*_*θ*_, *β*_*θ*_, *θ*, and *σ*, which result in maximizing the log-likelihood function *l*(*β*, *α*_*θ*_, *β*_*θ*_, *σ*) expressed by

(E.4) lβ,αθ,βθ,σ=−n2log⁡2π−∑1nlog⁡σaiθ−12∑1nwi−βθaiαθσaiθ2,

and in turn the linked homoscedastic regression model is

(E.5) $$\begin{array}{c} w_{i}=\beta_{o}{a_{i}}^{\alpha_{o}}+\epsilon_{i} \end{array}$$

for *i* = 1,2,…, *n* and with error terms, *ϵ*_1_, *ϵ*_2_,…, *ϵ*_*n*_, independent and identically distributed normally with common mean *μ* set to zero and having an invariant standard deviation *σ*. The response *w*_*i*_ above is normally distributed with mean function *μ*_*i*_(*a*_*i*_, *α*_*o*_, *β*_*o*_) given by

(E.6) $$\begin{array}{c} \mu_{i}\left(\;a_{i},\alpha_{o},\beta_{o}\;\right)=\beta_{o}{a_{i}}^{\alpha_{o}}. \end{array}$$

Corresponding log-likelihood function *l*(*α*_*o*_, *β*_*o*_, *σ*) becomes

(E.7) lαo,βo,σ=−n2log⁡2π−∑1nlog⁡σ−12∑1nwi−βoaiαoσ2.

It can be ascertained from Table 15 that estimates for the parameters *α*_*o*_ and *β*_*o*_ for raw data are very similar to *α*_*θ*_, as well as *β*_*θ*_, for the heteroscedastic regression model of (E.1) and (E.4), respectively.

Confidence intervals in Table 15 show some overlap. Therefore, it can be established that parameter estimates differ but just barely. This statement is supported by the estimated value of *ρ*, Lin's concordance correlation coefficient between projected and observed leaf biomass values. For both regression models, homoscedastic and heteroscedastic, the estimate was *ρ* = 0.972. Likewise, plotting the mean response curves *w*_*i*_ = *β*_*θ*_*a*_*i*_^*α*_*θ*_^ and *w*_*i*_ = *β*_*o*_*a*_*i*_^*α*_*o*_^ on a dispersion diagram of weight and area values reveals that curves corresponding to parameter estimates fitted considering heteroscedasticity or not are very similar (Figure 5). Nevertheless, the heteroscedastic fit clearly identifies for the variance a variation pattern that grows in a power function as leaf area increases. Indeed, Figure 15 shows remarkable differences in scatter patterns of dry weight residuals against leaf area for the heteroscedastic and homoscedastic models ((a) and (b), resp.). This can be ascertained from values in Table 6, unveiling that the heteroscedastic model is selected by most agreement indices.

Nevertheless, normal Q-Q plots of residuals of the fits of the heteroscedastic model shown in Figure 16 reveal that in spite of data cleaning we can be aware of a severe problem of heavy tails. This points to consideration of a different error structure from the normal one assumed here.

## References

[1] Huxley J. S. (1932). *Problems of Relative Growth*.

[2] Lu M., Caplan J. S., Bakker J. D. (2016). Allometry data and equations for coastal marsh plants. *Ecology*.

[3] Savage V. M., Gillooly J. F., Woodruff W. H. (2004). The predominance of quarter-power scaling in biology. *Functional Ecology*.

[4] Myhrvold N. P. (2016). Dinosaur metabolism and the allometry of maximum growth rate. *PLoS ONE*.

[5] West G. B., Brown J. H. (2005). The origin of allometric scaling laws in biology from genomes to ecosystems: Towards a quantitative unifying theory of biological structure and organization. *Journal of Experimental Biology*.

[6] Newman M. E. J. (2005). Power laws, Pareto distributions and Zipf's law. *Contemporary Physiscs*.

[7] Samaniego H., Moses M. E. (2008). Cities as organisms: allometric scaling of urban road networks. *Journal of Transport and Land Use*.

[8] Maritan A., Rigon R., Banavar J. R., Rinaldo A. (2002). Network allometry. *Geophysical Research Letters*.

[9] De Robertis A., Williams K. (2008). Weight-length relationships in fisheries studies: The standard allometric model should be applied with caution. *Transactions of the American Fisheries Society*.

[10] Echavarría-Heras H., Leal-Ramírez C., Villa-Diharce E., Cazarez-Castro N. R. (2015). The effect of parameter variability in the allometric projection of leaf growth rates for eelgrass (Zostera marina L.) II: the importance of data quality control procedures in bias reduction. *Theoretical Biology and Medical Modelling*.

[11] Echavarría-Heras H., Leal-Ramírez C., Villa-Diharce E., Cazarez-Castro N. (2018). On the suitability of an allometric proxy for nondestructive estimation of average leaf dry weight in eelgrass shoots I: sensitivity analysis and examination of the influences of data quality, analysis method, and sample size on precision. *Theoretical Biology and Medical Modelling*.

[12] Solana-Arellano E., Echavarría-Heras H., Leal-Ramírez C., Lee K.-S. (2014). The effect of parameter variability in the allometric projection of leaf growth rates for eelgrass (Zostera marina L.). *Latin American Journal of Aquatic Research*.

[13] Montesinos-López A., Villa-Diharce E., Echavarría-Heras H., Leal-Ramírez C. (2018). Correction to: Improved allometric proxies for eelgrass conservation. *Journal of Coastal Conservation*.

[14] Agutter P. S., Tuszynski J. A. (2011). Analytic theories of allometric scaling. *Journal of Experimental Biology*.

[15] Hui D., Jackson R. B. (2007). Uncertainty in allometric exponent estimation: a case study in scaling metabolic rate with body mass. *Journal of Theoretical Biology*.

[16] Packard G. C. (2013). Is logarithmic transformation necessary in allometry?. *Biological Journal of the Linnean Society*.

[17] Mascaro J., Litton C. M., Hughes R. F., Uowolo A., Schnitzer S. A. (2011). Minimizing bias in biomass allometry: model selection and log-transformation of data. *Biotropica*.

[18] Mascaro J., Litton C. M., Hughes R. F., Uowolo A., Schnitzer S. A. (2014). Is logarithmic transformation necessary in allometry? ten, one-hundred, one-thousand-times yes. *Biological Journal of the Linnean Society*.

[19] Thompson D. W. (1943). *On Growth and Form*.

[20] Smith R. J. (1984). Allometric scaling in comparative biology: problems of concept and method. *American Journal of Physiology-Regulatory, Integrative and Comparative Physiology*.

[21] Lovett D. L., Felder D. L. (1989). Application of regression techniques to studies of relative growth in crustaceans. *Journal of Crustacean Biology*.

[22] Bales G. S. (1996). Heterochrony in brontothere horn evolution: allometric interpretations and the effect of life history scaling. *Paleobiology*.

[23] Lagergren R., Svensson J.-E., Stenson J. A. E. (2007). Models of ontogenetic allometry in cladoceran morphology studies. *Hydrobiologia*.

[24] Sartori A. F., Ball A. D. (2009). Morphology and postlarval development of the ligament of *Thracia phaseolina* (bivalvia: Thraciidae), with a discussion of model choice in allometric studies. *Journal of Molluscan Studies*.

[25] Packard G. C. (2014). Multiplicative by nature: logarithmic transformation in allometry. *Journal of Experimental Zoology Part B: Molecular and Developmental Evolution*.

[26] Packard G. C. (2015). Quantifying the curvilinear metabolic scaling in mammals. *Journal of Experimental Zoology Part A: Ecological Genetics and Physiology*.

[27] Packard G. C. (2016). Relative growth by the elongated jaws of gars: a perspective on polyphasic loglinear allometry. *Journal of Experimental Zoology Part B: Molecular and Developmental Evolution*.

[28] Packard G. C. (2017). Is complex allometry in field metabolic rates of mammals a statistical artifact?. *Comparative Biochemistry and Physiology Part A: Molecular & Integrative Physiology*.

[29] Packard G. C. (2017). The essential role for graphs in allometric analysis. *Biological Journal of the Linnean Society*.

[30] Lai J., Yang B., Lin D., Kerkhoff A. J., Ma K., Bond-Lamberty B. (2013). The allometry of coarse root biomass: log-transformed linear regression or nonlinear regression?. *PLoS ONE*.

[31] Klingenberg C. P. (1998). Heterochrony and allometry: the analysis of evolutionary change in ontogeny. *Biological Reviews*.

[32] Nevill A. M., Bate S., Holder R. L. (2005). Modeling physiological and anthropometric variables known to vary with body size and other confounding variables. *Yearbook of Physical Anthropology*.

[33] Kerkhoff A. J., Enquist B. J. (2009). Multiplicative by nature: why logarithmic transformation is necessary in allometry. *Journal of Theoretical Biology*.

[34] Xiao X., White E. P., Hooten M. B., Durham S. L. (2011). On the use of log-transformation vs. nonlinear regression for analyzing biological power laws. *Ecology*.

[35] White E. P., Xiao X., Isaac N. J. B., Sibly R. M., Sibly R. M., Brown J. H., Kodric-Brown A. (2012). Methodological tools. *Metabolic Ecology: A Scaling Approach*.

[36] Ballantyne F. (2013). Evaluating model fit to determine if logarithmic transformations are necessary in allometry: a comment on the exchange between Packard (2009) and Kerkhoff and Enquist (2009). *Journal of Theoretical Biology*.

[37] Glazier D. S. (2013). Log-transformation is useful for examining proportional relationships in allometric scaling. *Journal of Theoretical Biology*.

[38] Niklas K. J., Hammond S. T. (2014). Assessing scaling relationships: uses, abuses, and alternatives. *International Journal of Plant Sciences*.

[39] Lemaître J. F., Vanpé C., Plard F., Pélabon C., Gaillard J. M. (2015). Response to Packard: make sure we do not throw out the biological baby with the statistical bath water when performing allometric analyses. *Biology Letters*.

[40] Strauss R. E., Huxley J. S., Thompson D. H. (1993). The study of allometry since Huxley. *Problems of Relative Growth*.

[41] Knell R. J. (2009). On the analysis of non-linear allometries. *Ecological Entomology*.

[42] MacLeod C. D. (2010). Assessing the shape and topology of allometric relationships with body mass: a case study using testes mass allometry. *Methods in Ecology and Evolution*.

[43] Mori S., Yamaji K., Ishida A. (2010). Mixed-power scaling of whole-plant respiration from seedlings to giant trees. *Proceedings of the National Acadamy of Sciences of the United States of America*.

[44] Snelling E. P., Taggart D. A., Maloney S. K., Farrell A. P., Seymour R. S. (2015). Biphasic allometry of cardiac growth in the developing Kangaroo Macropus fuliginosus. *Physiological and Biochemical Zoology*.

[45] Duffy J. E., Organisms., Duffy J. E., Thiel M. (2007). Ecology and eusociality in sponge-dwelling shrimp. *Evolutionary Ecology of Social and Sexual Systems, Crustaceans as Model*.

[46] Batterham A. M., George K. P. (1997). Allometric modeling does not determine a dimensionless power function ratio for maximal muscular function. *Journal of Applied Physiology*.

[47] Chave J., Andalo C., Brown S. (2005). Tree allometry and improved estimation of carbon stocks and balance in tropical forests. *Oecologia*.

[48] Niklas K. J. (1995). Size-dependent allometry of tree height, diameter and trunk-taper. *Annals of Botany*.

[49] Niklas K. J. (1997). Mechanical properties of black locust (Robinia pseudoacacia L.) Wood. Size-and age-dependent variations in sap- and heartwood. *Annals of Botany*.

[50] Packard G. C. (2017). Why allometric variation in mammalian metabolism is curvilinear on the logarithmic scale. *Journal of Experimental Zoology Part A: Ecological and Integrative Physiology*.

[51] Baskerville G. L. (1972). Use of logarithmic regression in the estimation of plant biomass. *Canadian Journal of Forest Research*.

[52] Newman M. C. (1993). Regression analysis of log-transformed data: statistical bias and its correction. *Environmental Toxicology and Chemistry*.

[53] Smith R. J. (1993). Logarithmic transformation bias in allometry. *American Journal of Physical Anthropology*.

[54] Duan N. (1983). Smearing estimate: a nonparametric retransformation method. *Journal of the American Statistical Association*.

[55] Koch R. W., Smillie G. M. (1986). Comment on “River loads underestimated by rating curves” by R. I. Ferguson. *Water Resources Research*.

[56] Manning W. G. (1998). The logged dependent variable, heteroscedasticity, and the retransformation problem. *Journal of Health Economics*.

[57] Zeng W. S., Zeng W. S., Tang S. Z. (2011). Bias correction in logarithmic regression and comparison with weighted regression for nonlinear models. *Nature Precedings*.

[58] Miller J. (1991). Short report: reaction time analysis with outlier exclusion: bias varies with sample size. *The Quarterly Journal of Experimental Psychology Section A*.

[59] Packard G. C. (2012). Is non-loglinear allometry a statistical artifact?. *Biological Journal of the Linnean Society*.

[60] Berg E. J. (1936). *Heaviside's Operational Calculus*.

[61] I-Kuei Lin L. (1989). A concordance correlation coefficient to evaluate reproducibility. *Biometrics*.

[62] McBride G. B. (2005). A Proposal for strength-of-agreement criteria for Lin’s Concordance Correlation Coefficient.

[63] Zeng W. S., Zhang L. J., Chen X. Y., Cheng Z. C., Ma K. X., Li Z. H. (2017). Construction of compatible and additive individual-tree biomass models for Pinus tabulaeformis in China. *Canadian Journal of Forest Research*.

[64] Zeng W. S., Tang S. Z. (2011). Goodness evaluation and precision analysis of tree biomass equations. *Sci Silvae Sinicae*.

[65] Zeng W., Duo H., Lei X. (2017). Individual tree biomass equations and growth models sensitive to climate variables for Larix spp. in China. *European Journal of Forest Research*.

[66] Akaike H. (1974). A new look at the statistical model identification. *IEEE Transactions on Automatic Control*.

[67] Anderson T. W., Darling D. A. (1952). Asymptotic theory of certain goodness of fit criteria based on stochastic processes. *Annals of Mathematical Statistics*.

[68] Bervian G., Fontoura N. F., Haimovici M. (2006). Statistical model of variable allometric growth: otolith growth in Micropogonias furnieri(Actinopterygii, Sciaenidae). *Journal of Fish Biology*.

[69] Brosowski B., Deutsch F. (1981). An elementary proof of the Stone-Weierstrass theorem. *Proceedings of the American Mathematical Society*.

